# A global picture of family medicine: the view from a WONCA Storybooth

**DOI:** 10.1186/s12875-019-1017-5

**Published:** 2019-09-12

**Authors:** Vincent K. Cubaka, Clayton Dyck, Russell Dawe, Baraa Alghalyini, Molly Whalen-Browne, Gabriel Cejas, Christine Gibson

**Affiliations:** 1 School of Medicine and Pharmacy, University of Rwanda, KG 11 Ave, Kigali, Rwanda; 2 Centre for Global Health, Department of Public Health, Aarhus University, Bartholins Allé 2, DK-8000 Aarhus C, Denmark; 3 Department of Family Practice, University of British Columbia, 5950 University Blvd #310, Vancouver, BC V6T 1Z3 Canada; 4 Discipline of Family Medicine, Memorial University of Newfoundland, Health Sciences Centre, Room 2743, St. John’s, NL A1B 3V6 Canada; 5 College of Medicine, Alfaisal University, Al Zahrawi Street interconnecting with Takhassusi St, Al Maather، Riyadh, 12714 Saudi Arabia; 6 Department of Family and Community Medicine, University of Toronto, 500 University Ave, Toronto, ON M5G 1V7 Canada; 7 Department of Family Medicine, University of Calgary, 2500 University Drive NW, Calgary, AB T2N 1N4 Canada; 8 School of Medicine, Universidad de Buenos Aires, Paraguay 2155, C1121ABG CABA, Argentina

**Keywords:** Family medicine, Family doctor, Family physician, Narratives, Stories, Qualitative, Motivation, Global, Value

## Abstract

**Background:**

Family Medicine is a novel discipline in many countries, where the motivation for training and value added to communities is not well-described. Our purpose was to understand the reason behind the choice of Family Medicine as a profession, the impact of Family Medicine on communities, and Family Medicine’s characterizing qualities, as perceived by family doctors around the world.

**Methods:**

One-question video interviews were conducted using an appreciative inquiry approach, with volunteer participants at the 2016 World Organization of Family Doctors conference in Rio de Janeiro. Qualitative data analysis applied the thematic, framework method.

**Results:**

135 family doctors from 55 countries participated in this study. Three overarching themes emerged: 1) key attributes of Family Medicine, 2) core Family Medicine values and 3) shared traits of family doctors. Family Medicine attributes and values were the key expressed motivators to join Family Medicine as a profession and were also among expressed factors that contributed to the impact of Family Medicine globally. Major sub-themes included the principles of comprehensive care, holistic care, continuity of care, patient centeredness, and the patient-provider relationship. Participants emphasized the importance of universal care, human rights, social justice and health equity.

**Conclusion:**

Family doctors around the world shared stories about their profession, presenting a heterogeneous picture of global Family Medicine unified by its attributes and values. These stories may inspire and serve as positive examples for Family Medicine programs, prospective students, advocates and other stakeholders.

**Electronic supplementary material:**

The online version of this article (10.1186/s12875-019-1017-5) contains supplementary material, which is available to authorized users.

## Background

Family Medicine (FM) is an academic and clinical discipline focused on the provision of continuous, comprehensive, coordinated and contextualized primary health care (PHC) for individuals, families and communities. FM considers biological, psychological, socio-economic, cultural and spiritual parameters and is not limited by age, gender, organ, system or disease. It incorporates prevention and health education within clinical care [1, 2]. Studies suggest that countries with strong PHC have more cost-effective healthcare systems, reduced health inequalities, and ultimately healthier populations [3, 4].

Many organizations support FM globally. These include the World Health Organisation (WHO), which emphasizes PHC as the pillar of the health system, and the World Organization of Family Doctors (WONCA), which aims to improve quality of life of the peoples of the world through promoting the values of FM [5]. The College of Family Physicians of Canada’s Besrour Centre also advances FM worldwide in its role as an international centre of collaboration between FM partners [6]. FM is relatively a new discipline in many countries and despite the high level support, it continues to present a wide spectrum of ground level challenges related to the variations in its scope and the heterogeneity in its practice within different global contexts [7, 8] .

The purpose of this study was to explore common perceptions and beliefs held by family doctors worldwide about their profession. By using an opportunistic approach to capturing their narratives, we hoped to identify their motivations and professional experiences, which would inform common strategies to advance FM globally. Such strategies may include student recruitment, engagement with institutions and non-FM peers, as well as engendering pride among FM colleagues. Through aggregation of these experiences, we organized this myriad of expressions into a descriptive narrative summary about the collective future of the discipline and the patients it serves.

The following study questions guided this exploration:

- What motivates family physicians to join FM?
- What is the impact of FM as perceived by family physicians?
- What traits do family physicians around the world share?

## Methods

### Study design

A lack of previous studies examining the feelings global family doctors have about their discipline led us to choose an exploratory qualitative approach that used data generated through short personal interviews of participants at an international conference. This approach allowed insight into participants’ perceived experience of FM in an unscripted and spontaneous manner. Seven international volunteer members of the Besrour Centre’s Narrative Working Group conducted the interviews. Four of the seven interviewers also contributed to transcription and data analysis.

### Study population

The study population was a convenience and snowball sample of family doctors participating in the 21st WONCA World Conference of Family Doctors in Rio de Janeiro, Brazil (November 2nd-6th, 2016). This event was purposefully chosen as it provided an international and heterogeneous cluster of informants. We included family doctors regardless of their current position and function. We only included those who speak English, French, Portuguese or Spanish and who consented and had time for a short video interview.

### Data collection

Data was purposefully collected from conference attendees, who were approached in person and invited to participate. Seven researchers performed the interviews using a standardised process:

1. Participants were given a brief explanation of the project and asked to sign an informed consent form.
2. Participants were asked to select and provide an answer 2–3 min in length to one of three questions from a provided instructions ‘menu’ (in English, French, Portuguese or Spanish) (see Fig. 1.).

**Fig. 1:**
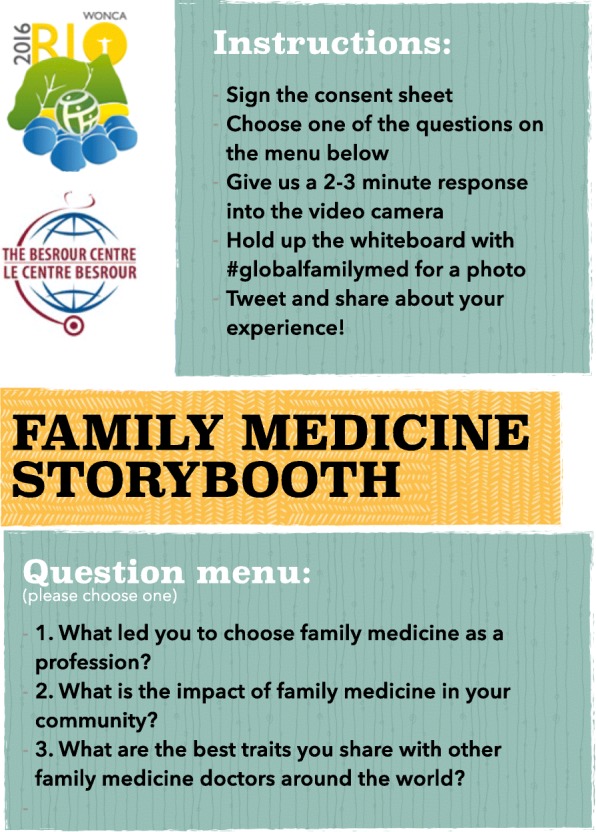
The ‘menu’ used during data collection (English version)
3. Responses were video recorded in participants’ preferred language.
4. Participants were invited to attend a session presenting the project’s preliminary findings on the last day of the conference (see Fig. 2.).

**Fig. 2:**
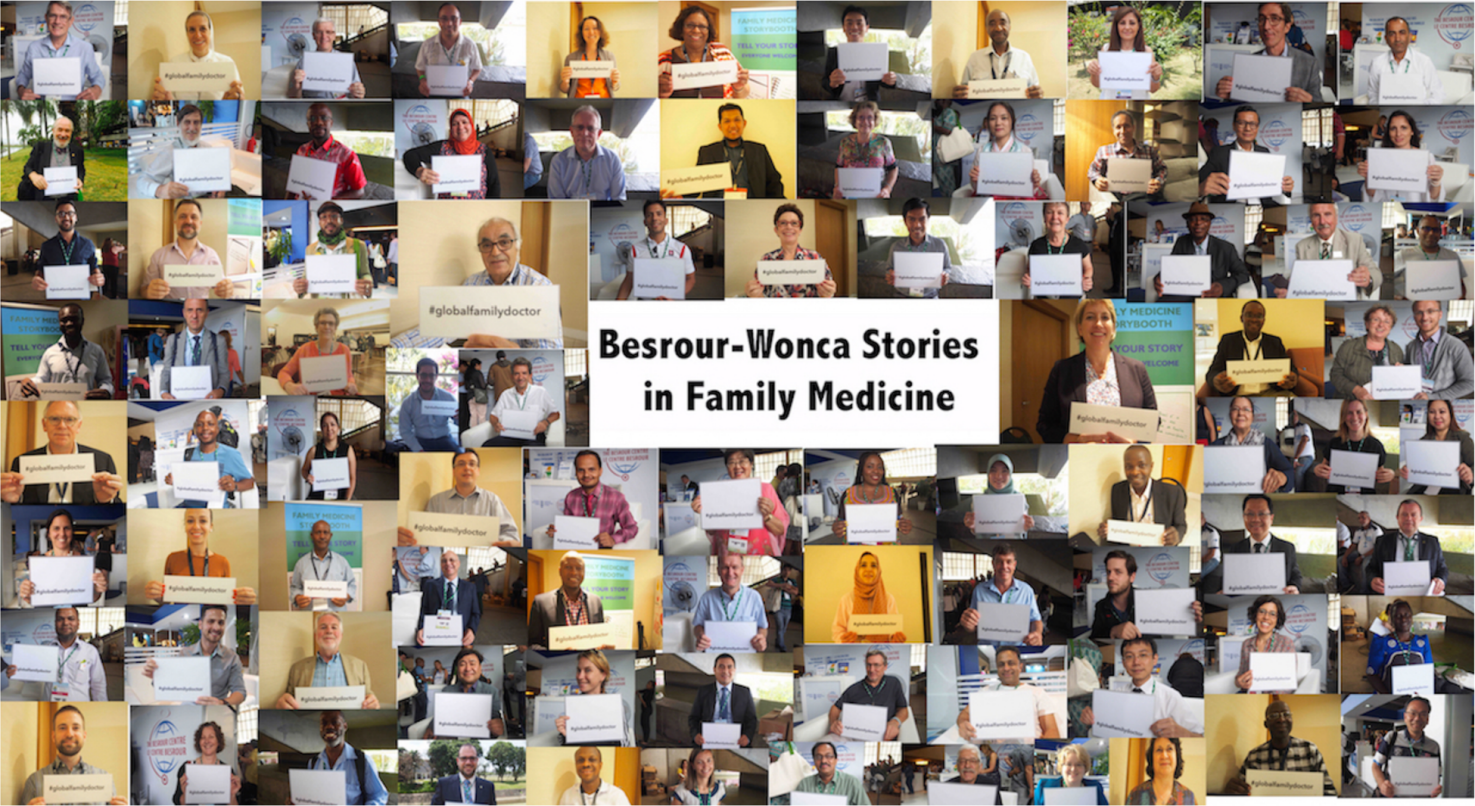
Participants of WONCA interviews photo collage

The authors transcribed these recordings verbatim; all non-English transcriptions (in French, Spanish and Portuguese) were translated into English (See Additional file 1). All transcripts were also validated against the digital video recordings.

### Data analysis

We applied a thematic, framework method [9] to our analysis coupled to an appreciative inquiry approach [10]. Seven researchers each reviewed 50–100% of the interviews each. They familiarised themselves with the material and generated individual codebooks by identifying major and minor themes from the transcripts. Two researchers (VCK and CG) discussed the seven codebooks and developed a master codebook that categorised emerging themes in main themes and subthemes. The master codebook was then reviewed by all researchers until consensus was achieved. Using MaxQDA qualitative data analysis software^8^, one researcher (VCK) systematically coded the text from all transcripts according to the master codebook and the study questions. This process facilitated the evaluation of connections between the emerging themes. Finally, the entire research team reviewed and attained consensus regarding the key findings.

### Ethical considerations

Ethical clearance was obtained from the Institutional review board of the University of Calgary (ID REB17–0185). Participation was voluntary and a consent form was provided and signed by each participant.

## Results

**Fig. 3:**
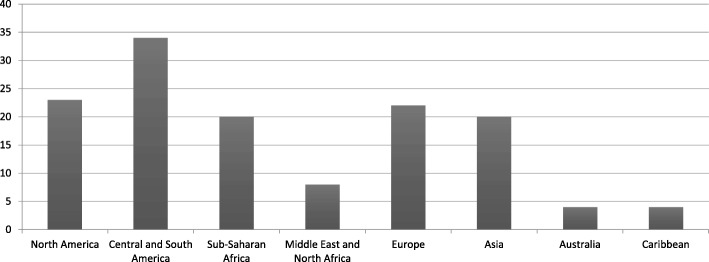
Interview participants by region

One hundred and thirty-five family doctors from 55 countries participated in this study. 84 were male and 51 were female. All the continents were represented, except Antarctica (Fig. 3).

Three overarching themes emerged from the material.

1. Key attributes of FM: what characterises the practice.
2. Core FM values: what practitioners value about their role.
3. Shared traits of family doctors: what characterises the practitioners.

Illustrative quotes are presented in Table 1.

**Table 1:** Main themes and illustrative quotes

Themes	Subthemes	Illustrative quotes
Key attributes of Family Medicine		
	Holistic care	1. *“When I graduated I wished to find a medical specialty that had a holistic view, that saw a person in his community, that contemplated his spirituality, his values, his beliefs, his principles; and moreover, that worked with a big implication in social issues […] it was where I chose Family Medicine”* Argentina 5
		2. *“This is our trade: family medicine specialists look at people globally and they address their psychosocial problems. This is a trait that is unique to us as family physicians.”* Lebanon 1
		3. *“I realized that what interested me the most was the patient in his entirety; it was not only an organ, it was not a system, it was really about who is in front of me, how the meeting between us will bring benefit to him.”* Switzerland 1
	Prevention and health promotion	4. *“Family medicine is very important, because not only does it focus on the illness but also on the prevention, and once you get to prevent we are avoiding that the whole family, patients, and the community lose in health and economy.”* Ecuador 3
		5. *“They are interested in continuing to work with us, not only on matters of health but also health promotion, so we visit them even when they’re not ill and we encourage them to come and see us […] So, we have seen significant growth in matters of raising up health conscience within the community we work with”* Kenya 1
	Comprehensive care	*6. “What moved me to family medicine is first of all the breadth of practice. It is very diverse, we see all sorts of patients. […] I was equally involved in the less favoured side than the rich side of the city.”* Canada 6
		*7. “I love to be a family physician because you are able to take care of the whole family”* Malaysia 1
	Universal care	8. *“I think that medicine should be dedicated to care for everyone’s health, especially for poor and needy people. Medicine shouldn’t be a business. That is why I chose Family Medicine as a profession”* Argentina 4
		9. *“I am a family doctor because I found family medicine a response to reduce the inequity that exists among health care users.”* Ecuador 1
	Patient-centred and people oriented	10. *“I […] meet family doctors from around the world and what strikes me is how we all share the same devotion to the people we care for and to the communities in which they are.”* Canada 8
	Bridging the family and community	11. *“You’re part of the community; you understand them, the context, the milieu, the whole ecology in which they live. You think about your patients all the time. […] We’re connected to our communities, connected to the people we work with, particularly in rural sites”* Australia 4
		12. *“I have been working in the same street with the same families for 42 years”* Belgium 2
		13. *“We can see the patient […] in the community where they live and I realized that it makes a huge difference because we can, by treating one patient, treat the whole family and reach in fact the whole community.”* Portugal 2
	Proximity	14. *“It [Family Medicine] affords primary care that is community centred; so for the most part, people who have a family doctor won’t have to access specialists, or hospital-based care and they can stay closer to their home and their environment.”* Canada 5
		15. *“Having these units of primary care gives better attention to people, allows physician to interact with the community, and makes medicine more accessible”* Paraguay 1
	Rural health	16. *“I believe that family physicians will benefit most people in my country […] especially in rural areas, so they can live better and cure their diseases.”* Indonesia 1
		17. *“I chose family medicine because at the time I took the training as a family physician it was not easy for people to provide full services at a very rural level, and family medicine turned out to be that specialty that could allow me at that time and up till now to provide broad based medical care, especially to people in rural areas where I come from.”* Nigeria 2
	Continuity of care	18. *“One of Family Medicine’s attributes is continuity of care, that is the most satisfying thing to the patient and brings more confidence to the doctors to deliver better care.”* Brazil 5
		19. *“I think what drew me to Family Medicine the most was really the continuity of care that we offer to our patients.”* Jamaica 1
	Drawn into Family Medicine	20. *“When I saw what he was doing, I was impressed. So I asked him ‘how can you do everything?’ and he told me ‘yeah, I am trained to do everything’. So I said, ‘what were you trained in?’ He said ‘I trained in Masters in Medicine, in Community Practice.’ At the time, because it had just been changed from Community Practice to Family Medicine. Then I said, ‘I think this is what I should do’”* Uganda 1
		21. *“Before I worked as a emergency doctor, but I changed it [to family medicine]. In Japan, elderly people was 25% […] so I thought it important for elderly people to manage complicated social, biomedical or psychological [issues]. They can’t be managed as emergency, so I chose it”* Japan 3
	Recognition	22. *“My patients are sometimes surprised that all of the clinicians there are family practitioners, not specialists. They’re “just a GP”. And sometimes it’s hard for them to get their head around the fact that “just a GP” will be advocating for them, and who holds their lives in their hands, and their hearts in their hands.”* Australia 1
		23. *“I had all sorts of prejudice with Family Medicine and I was always saying:* *I would never do it in my life. I hate what they do, they are “only” generalists working in a “health centre”. But gradually I convinced myself otherwise, and gave Family Medicine a chance. When I got into the residency in Family Medicine I realized how wrong I was”* Brazil 12
Core FM values Shared traits		
	Enthusiasm	24. *“So I saw skills, I saw attitude, I saw values, I saw passion, and I saw people being successful and happy at what they do”* Nigeria 1
		25. *“We really are very unified, very enthusiastic and very motivated.”* Canada 13
		26. *“Whatever be the place we work, the reality in which we are, we all share the love and passion to work for the community; and the desire to change things and to get better things in benefit of the persons who give trust to us.”* Ecuador 5
	Human encounters	27. *“As family doctors, we have values ​​that are very dear to us. First, there is respect, there is competence, compassion, but also integrity and also team spirit.”* Haiti 1
		28. *“I have human interests, so I am enjoying the stories I hear every day. It is an excellent job for the people who like people.”* Netherland 1
		29. *“I chose Family Medicine because what drew me to medicine in the first place is really the human stories that populate our discipline.”* Canada 9
	Patient-provider relationship	30. *“There’s more contact with the patient. They develop a long-lasting relationship, they say from the cradle to the rocker or from the womb to the tomb. And that’s the bedrock of family medicine.”* Ghana 1
		31. *“I feel like I have enormous good relationships over my very kind and nice community. And in that way it has given me the meaning in my life.”* Denmark 4
		32. *“The fact that I could develop a long-lasting relationship with my patients over the 30 years I was in practice (I saw children grow up, have their own children, and then bring their own children to me) gave me such a sense of being able to make a difference in the life of a family.”* Jamaica 1
	Enquiry	33. *“You’re challenged by their medical needs; you’re challenged by having to utilize the knowledge that you have in order to meet those needs. But also by having to put those medical needs in the context of who that person is: their beliefs, the community in which they live, their income that they earn, and being able to do that and keep them well over long periods of time is what I think family practice is all about”* Australia 3
		34. *“Life is awkward and messy, and wonderful and I think that’s what we share as family physicians: interest in the universe, curiosity, and willingness or hope to be able to assist or help and facilitate.”* Denmark 1
	Accountability	35. *“We know that we’re responsible for the patient, we know that she/he’ll always come back to us. So, when Family Medicine comes to a place like this, in a poor community, with people with no access to health services, it makes a huge difference in their lives, in every family.*” Brazil 11
	Caring for vulnerable	36. *“The reason why they choose Family Medicine [is] to help people even in low resource settings and the vulnerable population in their various communities.”* Canada 2
		37. *“So that’s probably it: the patients, the poor, and the passion for them.”* South Africa 1
	Happiness and fun	38. *“You need to have a sense of humour, you need to have fun. […] You can have fun in everything you do. So, a consultation, even the most difficult news can have a little bit of humour, a little bit of light-heartedness, a little bit of fun in it. Certainly you can enjoy yourself with your patients […] you find out an extraordinary amount of information.”* Australia 4
	Motivation	39. *“If I had to do it all over, I would choose Family Medicine again as it has been rich and exciting experience for me.”* Jamaica 1
		40. *“The reason why I wake every day in the morning and feel energetic and enthusiastic about going to my clinic is because it’s a very rewarding field. With each patient you get, you listen to a new story; you can never tire of what will be the next patients come complaining of.”* Saudi Arabia 1
		41. *“You know you’re making a true change in people’s life, and that for sure changed my life.”* Brazil 15
Shared traits of Family doctors		
	Specialist of common issues	42. *“After medical school I recognized that only family medicine would give me all of the skills that I will need to tackle most of the health problems in my community. And this includes communicable diseases and non-communicable diseases and mental health disorders and so on and so forth.”* Nigeria 6
		43. *“They had family physicians doing all kinds of different things: serving the communities that they lived in, and I really liked the idea that they were doing what was needed in their community and I thought that this was the job for me.”* US 2
	Adaptability and versatility	44. *“It encompasses everything I want to do as a doctor.”* Namibia 1
		45. *“Family doctors have a range of skills. A lot of them provide obstetrical care; some of them provide acute care, for in-patients or for emergency rooms. A lot of them do surgical procedures, academic research, […]. These are the kinds of things that when you put it all together, it’s the big picture of what the society needs. […] I think of a segment of physicians that are really fulfilling the need of the community that they serve. And I think that is what Family Medicine does globally.”* Canada 5
	Advisor	46. *“I think I share the interest in everyday life: how people live their lives, the sense of belonging in a community, this thing of being able to assist and facilitate people to live their lives, not to paternalize them but just to advise and hopefully be able to see them prosper and grow.”* Denmark 1
	Leader	47. *“For the fact that we are more community centred and so we are able to be community leaders and […] being an advocate.”* Ghana 1

### Key attributes of FM

FM was often presented as an evolving profession, providing a solution to common health problems and health system issues. Participants identified a number of key attributes of FM, including: comprehensive care, holistic care, patient-centred care, proximity with people and continuity of care. Many participants reported the provision of holistic care beyond the symptoms of a disease as a core principle of FM, taking into account mental and social factors as well as social determinants of health. This approach was attractive to many participants and influenced their choice to become family physicians (Quotes 1–3). Prevention and health promotion were also often highlighted as important in the practice of FM (Quotes 4, 5).

Participants recognized that FM provides comprehensive undifferentiated care to help all people (Quotes 6,7). Other participants declared that they joined FM because they believed in universal health care, in the reduction of health inequity and the promotion of social justice, particularly for underserved populations (Quotes 8,9).

Some participants described that family doctors are more connected and involved in the communities they live in than other physicians (Quotes 14,15). Continuity of care and helping patients through their illness journey were also considered attractive attributes of FM (Quotes 18, 19).

### Core FM values

Interviewed participants also focused on values that motivated them to join FM, shaped their attitudes, and guided their practice. These included empathy and compassion, sharing stories and human touch, enquiry and reflexion, accountability, team spirit, mutual respect, and preference for marginalised populations. Further values included the passion that drives family doctors to serve humanity and to build relationships that generate patient and provider satisfaction (Quote 24).

Many family doctors were enthusiastic about FM as a philosophy and practice, were passionate about their patients, and were ready to bring change and respond to the patient’s health needs (Quotes 25,26). They also valued listening to and sharing people’s stories, and advocating through these stories (Quote 28). They valued enquiry, curiosity, reflection and the variety of challenging situations that they deal with in their daily practice (Quotes 40, 41). Some participants thought that happiness and fun were important when doing their work, helping them to cope with the challenges they encounter (Quote 38). Many participants expressed their satisfaction with FM and some noted it provided a good work/life balance (Quotes 39–41).

### Shared traits of family doctors

The participants identified a number of shared traits: presenting themselves as *generalist* clinicians, helpers, facilitators, servants, advisers, friends, communicators, teachers, mentors, leaders, advocates, researchers, and scientists with multiple and different practice environments that require a high level of adaptability. They reported that they have a wide scope of practice (Quote 42, 44, 45). They also noted that they were prepared to be leaders who would be ready to influence people, communities and policy on health-related issues (Quote 47).

## Discussion

This study captured family doctors’ perceptions of their motivation to pursue a FM career, the impact of FM on communities and other shared traits of family doctors. FM attributes and values were often articulated around the concepts and principles of comprehensive care, holistic care, continuity of care, patient centeredness, strong patient-provider relationship and proximity with people. These concepts and principles were shared in varying degrees, depending on home region and the current state of FM development in their context. The examination of the narratives in Table 1 reveals that participants from countries where FM was well established tended to describe the direct impact of FM attributes and values while participants from countries where FM was relatively new were more hypothetical and tended to talk in term of wishes and aspirations [7]. We believe this difference in the narrative is part of the natural history of FM programs. Participants also emphasized universal care, human rights, social justice and health equity as important drivers of FM practice, as reported in another study [11].

FM attributes and values influence decisions around pursuing a career in FM. It has been shown that continuity of care as well as the broad scope of both pathology and patients, are some of the main motivators to join a FM career [11–13]. Studies show that early interest, personal experience of FM practice, positive mentors, a good work-life balance and communication influence the choice of FM career [11–16]. It has been reported that students who choose to join FM training programs are attracted by the unique patient-provider relationship and that income and prestige are not their first priority [14, 17, 18]. In other studies; variety, continuity of care and work–life balance were the top three reasons behind choosing FM as a career [19], with social dimensions of medicine, continuity of care and the satisfaction of successfully dealing with minor illnesses, being also elements of FM that particularly attract family doctors [20]. Perceptions from our participants were well aligned with these findings from previous studies, often done in high-income settings. However, it would be too simplistic to present the above motivators as the sole determinants of FM career choice. Indeed, the processes through which medical students choose a training programme are complex and depend on multiple personal and societal issues [11, 21]. Physician career choice is often determined by the balance between career preferences, the availability of training posts and career opportunities [20].

FM has been described as a challenging yet rewarding job and this was also reported by participants [22]. Physicians who develop careers in FM likely do so for reasons other than income and status [18]. As found in our study, family doctors are satisfied with their jobs due to the intrinsic qualities of their work, and work values seem to influence their job satisfaction. [23] A similar link has already been suggested in the literature; ‘because career commitment depends more on values than on any other factor, such as interests, abilities, and personality, values may be a more valid and reliable predictor of job satisfaction’ [24]. Therefore a focus on job satisfaction via factors that attract and retain family doctors may help future FM recruitment, practice and retention [25].

The field of FM continues to evolve around the world. For instance, in Ghana, FM is a relatively new speciality and the main reason of not choosing it is the lack of understanding among medical students [26]. Where FM is well established it is sometime overshadowed by more prestigious and lucrative specialities that tend to be favoured by academic, healthcare and social contexts [11, 12, 18]. In one study, students recognized that they are not exposed to FM during their training and therefore had little idea of what is involved in being a family doctor [27]. These are among the reasons why medical students may hesitate to become family doctors, making recruitment in FM difficult [16] and contributing to the scarcity of family doctors in many countries [17, 21, 28]. This consequently limits the development of primary health care systems [29]. Emphasizing the motivators to practice FM, as highlighted in this study, may attract, nurture and retain potential candidates with a predilection for FM. Indeed, engaging students early in their choice of career and increasing the presence of FM mentors might encourage interest in FM by improving students’ perception of FM as a career option and decrease the misperceptions that may negatively influence FM career decisions [14, 21, 30].

This exploratory study shows that family doctors around the world share attributes and core values of their profession. Further, the study shows that perceived FM traits, competencies and tasks tend to be diverse, and are often physician and context dependent. The results suggests that family doctors from different countries have a similar understanding of their discipline and of their careers as found in previous research [31]. Considering that FM is at different stages of development in the world, a question might be how FM attributes and core values are applied and achieved across regions. Also, as it has been already pointed out, we may ask again if these attributes and values really ‘bind family doctors together’ [32]. Future studies may help to answer these questions.

### Practice and research implication

Our findings may be used to advance and positively influence the global discourse on FM. The knowledge may help in guiding recruitment strategies and in accelerating professional identity formation for future family doctors. In-depth exploration of each of the highlighted motivators, values and success stories may help to deepen the understanding of FM on a global scale and find ways to attract those who will enjoy and sustain a FM career [17]. The identified themes may also be used for advocacy purposes especially in regions where FM is emergent.

### Limitations

Our findings should be considered in light of several limitations. This study is based on interviews from a convenience sampling of family doctors attending an international conference on FM. This entails a selection bias in our sample of family doctors who were probably enthusiastic about their profession, which may not represent the common view among family doctors and therefore we cannot claim saturation. Given that a visual capture of diversity was part of the desired outcome, we only included participants who agreed to use the photos and videos as part of our protocol. This may also have introduced a selection bias.

The majority of respondents were men (62%), which may affect the results. Since FM (and especially leaders who may be invited to a global conference) is still dominated by men in many parts of the world, these results can still reflect a real perception of the discipline. Participants were not asked for any other personal demographic data such as age, years of experience, etc., as we wanted to maximize the limited time available for interviews. This may have limited our interpretation of the data.

We used an appreciative enquiry approach with a focus on enabling factors in FM, which highlighted positive aspects of the discipline. However, this choice may have masked the existing tension about global FM described in the literature [7, 33].

The one-question interview approach may appear awkward for a qualitative exploration. Non-structured in-depth interviews with multiple prepared open-ended questions and spontaneous probes may have generated a richer dataset. However, as we expected participants at the conference to have a busy agenda, we anticipated this approach would not generate an adequate sample size. We therefore used the one-question interview approach and compensated its limitations by gathering a large and heterogeneous sample. Translation may be considered as a limitation. To minimize the risk of distortion of information translation and transcription was done by native speakers who are also fluent in English.

Of the three menu questions, the question about the impact of FM was rarely selected. Few studies have investigated this question and some have had counter-intuitive findings [34]. The study design did not allow us to influence choice of the question from the menu, so the relative lack of response to one of the queries could not be addressed. However, as all responses were coded for the same themes, we do not expect this to have a significant impact on our results.

Finally, as only two participants reviewed the findings, we cannot ensure their full internal validity.

## Conclusion

Family doctors from around the world shared stories and thoughts about their profession. We believe the main benefit of this study is the identification of attributes and values family doctors consider as important motivators in choosing their profession. These success stories may inspire and serve as role modelling for burgeoning FM programs. They may also help to design recruitment strategies, by focusing on factors that influence the choice of FM as a career. Expressed commonalities may foster the spirit of unity of global FM and boost common endeavours in improving global health through FM.

## Additional file

Additional file 1: Transcripts of the interviews. (DOCX 83 kb)

## Data Availability

Transcripts of all interviews are available as additional supporting files.

## Abbreviations

FM: Family Medicine
PHC: Primary Health Care
WHO: World Health Organisation
WONCA: World Organization of Family Doctors
