# Supplementary material for: A global picture of family medicine: the view from a WONCA Storybooth
Source: BMC Fam Pract. 2019 Sep 12;20:129. doi: 10.1186/s12875-019-1017-5 (PMC6740000; doi:10.1186/s12875-019-1017-5)
Supplement: Supplementary file 1 — Transcripts of the interviews. (DOCX 83 kb) [file 12875_2019_1017_MOESM1_ESM.docx]

ARGENTINA 1

I am a family doctor from Argentina, I practice family medicine in Buenos Aires and also occupational medicine, and I think that between both specialties and all doctors in the world, we share many values. Basically, doing a patient-centered medicine, and having some principles that guide our practice. To may helping people, putting us in their place, I think it is very important to make prevention practices, and to may especially to do empathy to understand the suffering of the other person. The ideal is that after medical consultation, the patient feels much better than when he/she arrived. Thank you very much.

ARGENTINA 2

I am a family doctor in Argentina, and I decided this specialty to be able to help and accompany the people in all the process of the life cycle, since they were born until the death, being with them in every moment, especially putting us in their place, in children as in adults, and something I always say I like very much to share life with them, when they grow up, got married, become parents, and have the possibility to share all those moments with much happiness, but also being with them in suffering moments, Making them feel better, holding their hands, and accompanying them to leave life in peace. That's the reason I chose family medicine.

ARGENTINA 3

I am (name), I am from Argentina, I am a first-year resident of family medicine, And I was asked why I chose family medicine...When I started university, I did not know the specialty, in my country, unluckily, this is not a well-known specialty, But I have always had interest in social things and in understanding medicine in a holistic way. And while I was learning, subjects as preventive medicine, communitarian medicine or having practices in primary care centers, I realized this started to interest me. I remembered that one teacher, that showed us the determinants of health of Mark Lalonde, that shocked me because they said it was more important health promotion, prevention, and environmental changes than medical interventions, so that made me re-thinks and reflects about where medicine was going to, and in what I would like to work, so that's why I chose family medicine and I am so happy with my decision.

ARGENTINA 4

Hello,

My name is (name) from Córdoba, Argentina.

Why did I choose Family Medicine as profession?

Well… I think that medicine should be dedicated to care everyone’s health, especially for poor and needy people. Medicine shouldn’t be a business. That is why I chose Family Medicine as a profession. I believe that in Argentina like all over the world Family medicine is the least recognized but the most sacrificed. And I think that in the future It’s going to be the most interesting to save the humanity. Good luck to everyone, I am pleased to be in Rio, see us in Seoul.

ARGENTINA 5

Well, my name is (name), I am from Argentina

I was born in Buenos Aires, I had the opportunity of studying in (Institution name), whit a biomedical model, with remains from military dictatorship of the 1982 year,

And, when I got graduated, I wished to find a medical specialty that had a holistic view, that saw a person in his community, that contemplates his spirituality, his values, his beliefs, his principles; and moreover, that worked with a big implication in social issues.

That led me to travel ... I have been working in Africa and in some poor regions of my country, and it was where I decided Family Medicine was what a passionate me and delighted me

And what was going to give me the opportunity to work from the humanitarian, social and community side I wanted

So, I did the residency in a rural region of my country, ending in 2012

And although I have had ups and downs

I still continue with this romantic thinking that the health is a human right, that has to be free, accessible, and of quality for all human beans of the world

And it is family medicine, my way to practice and give to my patients the love that I have for this specialty. Thank you very much.

ARGENTINA 6

The family medicine election in my case is related to the participaition with the comunity and the healthcare team inside of the family, the different age groups,

and because I believe that prevention and to give a continuos care to the family makes medicine more effective in accompaing, in illneses control and in family context wich is important to know.

AUSTRALIA 1:

I never intended to be a GP. I didn’t become one, in Family Medicine, until I was 37. And before that I worked in Africa and in Aboriginal health and I realized, while I was doing it, that the best way to gain the kinds of skills that I was using in these fields was to do family medicine training. So, I started very late. And at the time that I started it was the ?? days in Australia. So that was a, so in Australia it was managed mainly in terms of practice, it was a starting time, and a terribly sad time. Both sad and a time of, where general practice was of great use to people. So right from the beginning I had a sense that this was the right job and the best job, and immensely fulfilling job. For the rest of my professional life it’s never been boring. As opposed to many of my colleagues in other professions that doesn’t remain interesting forever. I’m now the big director of the refugee health service, and my patients are sometimes surprised that all of the clinicians there are family practitioners, not specialists. They’re “just a GP”. And sometimes it’s hard for them to get their head around the fact that “just a GP” will be advocating for them, and who holds their lives in their hands, and their hearts in their hands. I guess the delight of family medicine for me is that it continues to be something that rewards, intrigues, interests and is of service. Time: 1:48

AUSTRALIA 2

I’d like to tell you why I chose Family Medicine as a profession. I grew up in a very small town. I didn’t know many professional people, but I did very well in high school and I didn’t really expect to because it was such a small town. So, I looked around for something I would enjoy doing and I thought being a doctor would be a nice job in the community that I grew up in. So, I went off to university that was a long way from the town I was from. And I did medicine and ended up in a big town that I didn’t really like. Then I looked at the sort of work I could do in the place I grew up and that was Family Medicine, or rural medicine, seeing it’s where I’m from. I quite enjoyed it but it gets to be quite draining at times. So, it’s nice to come away for these conferences, like WONCA. Total: 1:15

AUSTRALIA 3

Oh, you’re not going to ask the question, my name is (name) from Australia and I’m going to answer the question of what are the best traits that you share with other Family Medicine doctors from around the world. I think the trait that I find I like the most is the fact that I love the comprehensive nature of family practice. I love the fact that we deal with patients over a long period of time; and they become your friends as well as your patients. And you’re challenged by their medical needs; you’re challenged by having to utilize the knowledge that you have in order to meet those needs. But also, by having to put those medical needs in the context of who that person is: their beliefs, the community in which they live, their income that they earn, and being able to do that and keep them well over long periods of time is what I think family practice is all about. Total: 1:12

AUSTRALIA 4

Around the world the more I’ve come to conferences the more struck I am that we have much more in common than we have different. The first thing is all family doctors love inquiry. They’re interested in their patients. They’re curious. So, you’re curious about the person in front of you, not just, you know, their condition, why they present that day and then why or what they’ve done, but their lives. It’s so interesting to find out about our patients’ lives, their families, and certainly in rural communities patients love that. You are part of the community; you understand them, the context, the milieu, the whole ecology in which they live. You think about your patients all the time. So, the last thing I did before I came to this conference was actually to call a patient on a Saturday afternoon to check he was okay. Because he had had a problem that had taken a while to settle down. His boss was giving him a hard time at work and I was really relieved to find out that was okay.

The second thing I think about is connection. So, we’re connected to our communities, connected to the people we work with, particularly in rural sites, and of course connected with our staff and the teams that we’re part of. I remember vividly in rural practice the librarian used to drop books off for me at the practice when she came for consultation or to pick up her script, because she knew I didn’t have time to get there during the hours that the library was open. I remember the patients not disturbing me on the weekend. So, they were connected to me, they knew you were entertaining or had a party, so they’d stay at home and not disturb me, and ring me that night or the next day. And I’d actually say, “well, it’s okay to disturb me.”

The final thing I think about, a trait that other family doctors share is having fun. You need to have a sense of humor, you need to have fun. My job now is very much in education and we have a lot of fun when we teach. But you can have fun in everything you do. So, a consultation, even the most difficult news can have a little bit of humor, a little bit of lightheartedness, a little bit of fun in it. Certainly, you can enjoy yourself with your patients. In Australia we spend a lot of time excising skin cancers. And you just chat to your patient while you’re doing that, you’re filling the time and you find out an extraordinary amount of information, but you have fun while you’re doing it. So, they’re my messages to family doctors across the world. Thank you very much.

BANGLADESH 1

This is Dr. (name) from Bangladesh. I represent [name of institution?] from Bangladesh. Bangladesh has Bangladesh College of General Practitioners and Bangladesh Academy of Family Physicians, two institutes […?] . I am serving as a family medicine. You know Bangladesh is a hugely populated country, that means [? seventeen whole/rural?] people we have so many problems, most of the people live in rural areas where specialist doctors are not so much available. Most of the people are lived in rural area, but our ah most of the doctors are staying in urban area. So rural people are really need doctor – qualified doctor – and rural ah country like Bangladesh ministered by family physician well and it is very necessary to serve the whole country by family physician. It can ah possible whole family can be treated by one doctor so that’s why we’re developing […] we must develop family physician.

BELGIUM 1

Ok, well I started my studies in medicine because I actually wanted to be, have human contact. I actually am from the first sixty-eight group of people so we are sort of utopists, and we really wanted to change the world. So, my story is actually that when I was a student, I was upset by the very oriented to…the basic sciences training I got, so we started a social cultural movement of students actually claiming for more work on the human aspects, counselling, the social dimensions of care.

And so, after my studies actually I was elected to go into internal medicine and I did that for four years because I thought we were not properly trained well enough. But after four years of medical medicine…internal medicine I’d seen it and I didn’t really want to care for the people, and so I actually started a parallel primary care practice and research in community health. And so from community health studies I actually went to health behavior change studies, I went to dealing with problems behind the stories, which are alcohol, social inequality and actually I have created several groups internationally around dealing with alcohol issues, dealing around mental health and now running the, actually, the group on family violence.

And I think the last years of my career I actually spent to help the young doctors see actually the social dimensions of care and the family dimension of care and learning to skip off the medical specialist oriented technical model of really counselling with the person and the family are actually encountering them. Total: 2:16

BELGIUM 2

Hi, my name is (name) and I am a family doctor in Belgium. I have been working in the same street with the same families for 42 years and I am also a researcher in family medicine. So, I will try to answer the question: Why did you choose family medicine?

In the 1970s, family medicine did not exist. One became a general practitioner if one did not want to compete to become a specialist. Since I do not like chefs, I do not like hospitals, and besides I did not have much money, I settled in an economically fragile neighborhood and started working. I realized that general medicine was not what I had been taught. That's probably what made me go on. After that I traveled abroad. I was in countries where there was even less than my country. I have learned millions of things from people. I came back and started a group medicine, which in the 80's was almost forbidden. And for 40 years, I have been working in group medicine. I just changed my business model, because my group decided to switch to capitation funding, which frees me from being charged by patients because they are sick. It has always disturbed me ethically to be paid to care for the poor. Here I am now very happy to be paid monthly and to be able to develop in a different way the concept of family medicine in the same neighborhood where I have always been.

BRAZIL 1

My name is (name) and I’m a family doctor. I have chosen family doctor (because family) because it’s a good specialty here in Brazil. It’s more comprehensive (and we can) and I couldn’t choose the heart, the lung, the head (ah) so I have chosen my specialty where I can study all the people.

It was interesting because it’s a specialty that was beginning eleven years ago, so I’m very happy (for) to choose.

0:47 So family medicine, here in Rio, is growing up very, very fast. We started with 1,000 covered by (***good??) health care, and now we have four millions of people in eight years.

1:05 So this specialty here in Rio is becoming very important. Now we form for almost 200 of new family doctors putting here in our residency program. So, this is the biggest and most important residency program here in Brazil. Total 1:32

BRAZIL 2

Hi, I decided to go into Family Medicine because when I got into Medicine, I also studied religious studies and entered the congregation. The goal of the congregation was to send me into mission. Family Medicine was a great option to be able to have an opportunity to do many things. It’s a world of opportunity – you could go into management, help in the hospital, and also in the communities. It ended up that through Family Medicine, when I got to Brazil, it opened many doors. Family Medicine was just starting in Brazil, so I helped to start some of the first communities for family health in San Paulo. Now we have medical faculty, which is based on Family Medicine curriculum, and it opened great doors. We are also now trying to implement it in other African countries because Family Medicine is about helping people have more health. It’s about caring for people; people going into Family Medicine are great people. Thank you.

Total: 1:16

BRAZIL 3

Hello, I’m (Name), I’m a Family Physician in São Paulo, Brazil and I chose Family Medicine because it is for me the best way to approach our population, providing access and coordination of care, guaranteeing continuity and comprehensiveness of care, based on social justice and social development. For me, the main impact of Family Medicine in my community is the possibility to provide a high-quality treatment, effective and efficient. That’s why I chose Family Medicine, because we can share and talk about all these possibilities around the world in this process (?), - Thank you.

Total: 0:55

BRAZIL 4

What I believe that is the main impact of Family Medicine in the community where I work. First of all, I must say that the community that I work with, is a Family Medicine residency program. I see that Family Medicine can transform the lives of young doctors and medical students (people that always thought about medical specialties in hospitals or very specific medical specialties) showing them a whole world of opportunities in front of them, with many nice and rewarding things, such as manage chronic conditions, manage undifferentiated problems, manage situations encompassing both biomedical, emotional and social issues. I think the diversity of subjects surrounding the scope of Family Medicine is, for a new doctor starting the residency program, the most enchanting and delightful thing, as well as the broad spectrum of action that a Family Physician has, far beyond the guidelines and the treatment of single conditions. That’s the most rewarding thing I see in my community of Family Medicine residents.

Total: 1:38

BRAZIL 5

0:04 - I choose Family Medicine after 2 years practicing primary care as a generalist

0:10 - before starting my residency.

0:13 - When I tried to find a medical specialty for me

0:16 - I found myself worried that

0:19 - any patient had several ill health issues,

0:24 - each one could be linked to another,

0:26 - or linked to his socioeconomic conditions and his family.

0:31 - I realized that everything could influence the treatment -

0:35 - the way that each patient

0:38 - understands his own illness and his own health condition.

0:42 - I saw in family medicine a better way to approach this scenario

0:47 - feeling fulfilled when I treat my patients.

0:56 - I could also experience what the continuity of care could bring to my work,

0:59 - having more contact with my patients.

1:04 - I could really see the outcomes of my practice and the impact that I could have in my patients' lives,

1:11 - and it can only be measured, most of the time,

1:15 - in the long run.

1:17 - One of Family Medicine’s attributes is Continuity of Care,

1:21 - that is the most satisfying thing to the patient

1:28 - and brings more confidence to the doctors to deliver a better medicine.

BRAZIL 6

Hi, my name is (Name). And I’m very happy to be here talking to you about why did I choose to be a family physician.

And I choose to talk about three reasons.

First reason is I love people, simply love people. I believe that’s the main reason.

Second reason is one I’ve been sharing with residents I work with. And that’s about being my own self. Not needing to be someone else or act someone else’s shoes. I can still be myself and be a family physician.

And the third reason is something I’ve learned through the years doing my work, which is being constantly challenged.

And I believe those are the main reasons why I choose to be a family physician.

BRAZIL 7

0:02 - I chose Family Medicine as a way to be closer to my community,

0:07 - understanding its ill-health issues,

0:11 - being ready for any health action that would be necessary to do.

0:15 - I believe that working on prevention, closer to my community,

0:21 - I’ll reduce the rates of admissions in hospitals and emergency rooms

BRAZIL 8

0:04 - I chose Family Medicine because

0:07 - it’s a medical specialty where I can deal with every life stages

1:13 - Starting from prenatal care, first years of life, until chronic conditions in the elderly.

0:24 - Beside being a doctor, I am an actor and a singer.

0:30 - And I can learn a lot in Family Medicine about people,

0:39 - Having a broad view on the human being and its life stages.

0:42 - It’s like a laboratory. When an actor creates a character he works on it as in a laboratory.

0:49 - In Family Medicine I can fell myself helpful as a doctor

0:53 - and it gives me back the ability to better understand the human being and all sort of feelings and emotions.

1:04 - It’s fullness

1:06 - And a Family Physician has to be a good clinician, as well, well trained and skilled.

1:11 - so I fell myself fulfilled, using a biopsychosocial approach,

1:17 - it helps me as an artist and it enables me to be better clinician.

1:20 - That’s why I chose Family Medicine

BRAZIL 9

0:00 - Family Medicine has a huge impact on the population that I deal with

0:08 - Most of the health problems we can solve easily

0:16 - coordination of care is a very important aspect that we can also provide.

0:20 - we always try do give a comprehensive care with a biopsychosocial approach.

0:33 - with that, I believe that the patients can be closer to their health team, having a better treatment to their health problems

BRAZIL 10

0:01 - Why I chose Family Medicine?

0:05 - Because at the medical school I felt uncomfortable dividing the patient by organs and systems.

0:12 - In Family Medicine, we see a patient as a person

0:16 - It’s a doctor that is not only concerned about prevention,

0:20 - but with coordination of care, with the social context of the patient,

0:26 - his family structure and how are they dealing with the health problems as well.

0:32 - we’re present in every life stage of a patient and that is extremely rewarding.

0:37 - To perform a prenatal care of a woman, see the baby growing until the day she will be a woman too,

0:44 - and then follow her prenatal care when she got pregnant

0:47 - It’s delightful

0:49 - It’s the return of a doctor that sees the patient in a global way.

0:53 - I notice that every people - rich and poor -

1:03 - miss a kind of doctor that can see one patient, bring together all his problems,

1:08 and then treat the person beyond the diseases, not only the body.

BRAZIL 11

0:01 - My name is (Name).

0:05 I chose Family Medicine because I couldn’t choose any medical specialty

0:11 - I like everything and I like people a lot.

And I’d like to be a doctor as in Noah Gordon’s novel “The Physician”,

0:20 - So… I thought it could be my way

0:25 - At my residency in Ribeirão Preto at USP it was like that: We saw the patient as a whole,

0:34 - Indeed, Unfortunately, we don’t have to much time to it and

0:38 - Routine and politics forbids us from seeing people this way

0:43 - and that is what I share with all Family Doctors:

0:47 - The willingness to help people, seeing the patient as a whole

0:53 - and make a difference in their lives.

0:57 - The impact of Family Medicine on the population that I deal with,

1:03 - Guaianazes, São Paulo East side, an extremely poor community, full of violent crimes

1:09 - I see that looking at people, willing to help,

1:16 - willing to do something to them,

1:18 - That is something they are not used to.

1:21 - a Family Physician approach this way,

1:24 - but the other specialties don’t

1:27 - They just want to discharge the patient out of their office,

1:32 - But we know that we’re responsible for that patient, we know that he’ll always come back to us.

1:37 - so, when Family Medicine comes to a place like this,

1:40 - in a poor community, with people with no access to health services,

1:44 - it makes a huge difference in their lives, in every family.

1:47 - It’s very beautiful!

BRAZIL 12

0:00 - I’m a Family Physician in Brazil.

0:05 - My story with Family Medicine started at the Medical School,

0:12 - In my rotations in Primary Care,

0:16 - But I had a lot of prejudice about Primary Care.

0:23 - I wasn’t so interested in Primary Care at that time,

0:29 - but there was something that always got my attention: the patients!

0:34 - During my medical studies, I was very interested in the clinical content.

0:40 - At the third year of Medical School I joined a league for intensive care studies

0:47 - And at the fourth year I became the president of this league.

0:52 - Therefore, I was really interested in doing Intensive Care as a professional career.

0:57 - But, when I've started my clerkship at the fifth year, I started doing all the rotations.

1:09 - I went to the Intensive Care rotation and I got frustrated,

1:14 - because I couldn’t have a relationship with the patients - and that’s what I liked the most.

1:22 - In my third year I kept contact with a teacher that taught me about the importance of

1:26 - focusing the care on the patient.

1:33 - That draw my attention, but I wasn't able to put it into practice.

1:35 - Then, in my fifth year I felt that something was missing in my clerkship, but I couldn’t really notice what was missing,

but I felt that learning only clinical contents wasn’t enough.

1:44 - And then, in the sixth year I got lost, trying to figure out what specialty to pursue.

1:51 - Everything that I did during those years was focused on Intensive Care, but it was not one of my options anymore.

1:57 - I ended up getting lost!

2:02 - And then, by destiny or fate, - actually I think it was a good fortune -

2:08 - in the middle of my sixth year I went to Pará (in the Amazon Forest), to do a rotation on a riverside community.

2:17 - I started to understand that we don't need too many things to do a good medical practice.

2:25 - You need to pay attention to the patient, trying to understand his environment

2:30 - and try to find ways to be helpful for him and his health.

2:37 - That struck me a lot!

2:39 - I was so used to work with technologies everytime that I needed to make a diagnosis,

2:47 - And I realized that, with so few resources available, we could do a lot.

2:51 - Patient went back to their homes satisfied.

2:54 - Then I called one of my teachers, who was a Family Physician,

2:59 - And he motivated me a lot to do Family Medicine.

I had all sorts of prejudice with Family Medicine and I was always saying:

3:07 - I would never do it (family medicine) in my life.

3:10 - I hate what they do, they are “only” generalists working in a “health center”.

3:15 - But gradually I convinced myself otherwise, and gave Family Medicine a chance.

3:19 - When I got into the residency in Family Medicine I realized how wrong I was.

3:25 - Today, after 5 years (2 in the residency and 3 working as a Family Physician),

3:35 - I can see how wrong I was and,

4:42 - if I could change, I think I can change other people as well.

3:45 - I can say that I’m a Family Medicine enthusiastic.

3:49 - Every time I try to talk about the importance of Family Medicine.

3:55 - That’s my story - a little bit long - but that’s the experience I lived:

4:02 - It is worth fighting against your own prejudice, and it is worth to make a better Family Medicine.

BRAZIL 13

0:03 - What I share with other Family Physicians

0:06 - from São Paulo, Brazil or the world

0:09 - is the interest on really taking care of people,

0:13 - not only following a guideline, but doing the best for my patient.

0:21 - It doesn’t mean necessarily that we’ll give medications, 0:26 - but also welcome his demands,

0:30 - trying to understand his reality, his family and his community.

0:34 - For us to go together towards a better treatment

0:39 - and for the best life that he could have.

0:41 - For me, that means to be a Family Physician

0:44 - And I learnt that here at WONCA

0:47 - That’s what we want: the best for the person that we’re treating.

BRAZIL 14:

Ok, well I choose family medicine because it was an old dream. I was a child when I started to think about it and I like to help people to know themselves and to prevent what they, they feel. Kind of, if you feel some, some pain, you must treat it. But if you prevent the pain you won’t need to treat it. And, when I was five, when I was five, I started to learn about children and how to take care of them, and how to breastfeed them, and then I decide I want to teach the others to take care of the children, and everybody. First of all, I was a pediatrician and it was a very good experience too. But my dream now is complete because I am a family doctor. Is it kind of? (background noise)

Time: 1:18

BRAZIL 15

Hi, my name is (name). I’m a rehabilitation doctor for 26 years and I was tired of offering patients technology that they didn’t care about, they didn’t want and they wouldn’t use. Then I moved to family medicine four years ago, working in maybe the highest, most violent place in Sao Paolo, Brazil, which is called Capao Redondo. Uhm, and there I feel every day I get younger and younger because I can go home and sure that I changed people’s life or better, every single day for the last 13 years. I know I did that. And not only people but also the community because we’re with the families and also the community. So, we can… When I began we had 83 bars and no market. People had to walk 4, 3 km to buy food. And now, there are 3-4 places where they can get food. In the neighborhood they have exercise academies. And so, you know you’re making a true change in people’s life, and that for sure changed my life. Also, we had a dump, a trash dump, a big thing that became a field for exercise. People practice there. We quadruplicated the rate of tuberculosis diagnosis. For the past ten years we don’t have one single person dying of tuberculosis in my area. And also, no mortality for maternal and infants. Thank you. Time: 2:01

CANADA 1

So, when I entered medical school, like most medical students, I did not have a clear direction at the time. I knew I loved people and I knew I liked to be stimulated intellectually, so the first couple of years as I went through medical school,

0:24 “I tried things and found I liked just about every discipline that I came across. Most importantly, I fell in love with the people I was interacting with, including other physicians and colleagues.” 0:38

So, after several years, I had the opportunity to spend three months in a busy family practice in Thunder Bay, where I got to see full-spectrum Family Medicine: all of the challenges of the intellectual stimulation, but probably

0:52 “most importantly the reward of having continuous relationships with patients over extended periods of time.” 1:00

The mentor that I had was really quite exceptional and he asked me to join his group. From that point on, my career in Family Medicine was pretty well decided upon. As I progressed through all the things that I saw and experienced in the three months that I was with him between my second and third years of medical school proved to be true, definitely proved to be a challenging intellectual academic enterprise, but more importantly, on reflection,

1:33 “as I finish my career after 37 and a half years, the reward of being involved with patients for that period of time and the privilege of being involved in their lives and secondly being involved in communities and the privilege of being involved in helping to morph communities from the health care perspective really has proven to be every bit as rewarding as I hoped it would be at the beginning.” Total 1:59

CANADA 2

What led me to Family Medicine? I really don’t remember. (because I) Since I was 14 years old, I knew that I was going to be a Family Medicine doctor. I went to medical school early, when I was 18, and I was a physician at 23. I practiced for a bit and then moved to Canada and I changed from general practitioner to a specialty, Family Medicine. It was a kind of nice switch because I saw quickly the relevance of Family Medicine and the impact of a Family doctor in a family.

00:58 “One other thing that I like about Family Medicine is that you never know what is walking through the door, you never get bored, you never know everything. You know in a specialty, if you’re an ophthalmologist, you only see just eyes, here, you never know – just everything can walk through the door, from a new born baby to a 100-year-old great-grandfather or a pregnant woman – so you never get bored. Total 1:35

CANADA 3

Thank you very much for that question. Having met some of the Family doctors from around the world, especially here at the Besrour conference during the Family Medicine forum, and other settings, I think

0:27 “one of the traits that stands out among all Family Medicine doctors I’ve met and I share with is passion. They have passion for improved and whole patient centered care and I think this is very important because one of the central priorities of every family physician should be advocacy and having a patient centered approach to care.” 0:57

I also find 1:02 “another trait that stands out to me is the genuine love for Family Medicine and the reason why they choose Family Medicine and to help people even in low resource settings and the vulnerable population in their various communities as well.” 1:25 I think another thing that stands out is integrity and advancement of Family Medicine globally among these physicians that I’ve met. To expand on that, most of them have an inherent desire to promote Family Medicine. They say not just in their various countries, but have a dream that Family Medicine will be a global phenomenon and will be practiced in various countries as well and I think this is a very exciting trait and I hope this is realized sometime in the future. Total 2:03

CANADA 4

Some of the best traits about Family Medicine are, on the one hand it’s about the eagle that’s flying high in the sky and has to take in the whole landscape. So, it’s your hub, it’s the one who sees all the players at the game and coordinates. It’s also like the octopus in the sea which, with seven arms (I know he has eight), but with seven of them, he reaches out and sees the players that are needed and pulls them in and with that eighth arm reaches to that gap and then plugs it himself. Total :38

CANADA 5

I think Family Medicine adds a lot of value to my community in Canada. Firstly, it affords primary care that is community centered; so, for the most part, people who have a family doctor won’t have to access specialists, or hospital-based care and they can stay closer to their home and their environment. Family doctors provide continuity, so they understand not just the person, but also the context of the illness that they are experiencing. Family doctors have a range of skills. A lot of them provide obstetrical care; some of them provide acute care, for in-patients or for emergency rooms. A lot of them do surgical procedures, academic research, consulting for things like medical exams for pilots or scuba divers. These are the kinds of things that when you put it all together, it’s the big picture of what the society needs. So, when I think of what happens in Canada, I think of a segment of physicians that are really fulfilling the need of the community that they serve. And I think that is what Family Medicine does globally.

Total: 1:21

CANADA 6

What moved me to family medicine is first of all the breadth of practice. It is very diverse, we see all sorts of patients. In my general practice in Montreal, I was equally involved in the less favored side than the rich side of the city. During the day, I could see the difference between the two kinds of practice. It is a practice that is quite broad and that is always captivating. There is no routine in everything I do every day: I work in the ER (emergency), after that in the office, and after that in private clinics. So, it's quite broad as practical.

CANADA 7

I’m thinking about what we family doctors have in common – what makes us both similar to each other and different from everyone else. 00:11 “I think at the base of it, is a taste for gossip. I think we tend to be the kind of busybodies that love to know everybody else’s’ business. So that means the more generations of any given family we have, the better we like it.” 00:28 We get to talk about Aunt Vera with cousin Ethel, about the grandchildren with the grandparents, and become somebody that is important in their lives. I think in some ways what validates us is the degree we become important, even though we don’t know how important we are to the people we take care of. So that’s the sort of ego-stroking aspect of it. The generative aspect of that is I think that all of us as human beings need people like family doctors, who know lots about them, share lots of their secrets, but who also demonstrates caring about them in important ways. The times in my practice life that have been important to me, have been memorable to me, have been the times when patients have said to me “Do you remember when we talked about x, y or z” or “Do you remember when you talked me through this or that problem – do you know how important that was?”. For me, that was one of 20 encounters I had that day; for them it was something that was special and important and being reminded of that I think takes us back to what it’s for and takes us back to what we both need and what people need us to do. Total 2:00

CANADA 8

I have had the opportunity during my nearly 40 years of practice to travel a lot and meet family doctors from around the world and what strikes me is how we all share the same devotion to the people we care for and to the communities in which they are. It may seem to be said and repeated, but it is something that is quite fundamental, partly because of this approach of the whole person. Finally, our approach to diagnosis and our approach to problem solving is very similar, even though sometimes different health problems differ across countries. So, for me it's something that always affects me when I have the chance to meet colleagues who come from elsewhere.

CANADA 9

“I chose Family Medicine because what drew me to medicine in the first place is really the human stories that populate our discipline. I’m interested in getting to know people, getting to know them over long periods of time, and understanding not just their health in the most narrow sense of the word, but really what makes them tick, what keeps them well.” 00:25

I like working with them to make them well and better and really Family Medicine is the only discipline that allows us to do this over long periods of time and to the level of depth that I was hoping to experience in my professional life. Total 0:39

CANADA 10

I think that one of the best traits that I have as a family physician that I think I share with many people here at WONCA is my joy in my relationships with my patients. I’ve had a clinical practice with a group of people for the past 16 years and I’ve come to know them well through their ups and their downs and they have opened their lives in terms of healthcare. We’ve established a wonderful relationship of which I am really proud of achieving and really happy for. So, I think that is one of the traits I see that most of the physicians if not all of the people that I spoken with here at WONCA.

Total 0:37

CANADA 11

I graduated from medicine 40 years ago and when I try to remember why I became a family doctor, it’s difficult. But my recollection is that going through Medical School you didn’t really know you had to become a certain kind of a doctor. And then I went to Bella Coola, in northern British Columbia, and I was in a small community of 2,000 people with 2 other family doctors. They did surgery, anesthesia, obstetrics, looked after the indigenous population, in a beautiful part of Canada and I thought: this is what I want to do. I realized I was attracted to the scope of the practice, to the ability to really make a difference in people’s lives. But also, from a personal point of view, it was the most challenging profession I could find: it was intellectually challenging, it was physically challenging, mostly emotionally challenging, and what challenged me most in terms of trying to make a moral difference in this world. Trying to find a way to improve the lives of people I was trying to look after. And I’ve never regretted my choice. It’s been a wonderful profession, and continues to be a wonderful profession, and I’m so glad I’m a family doctor. Total 1:20

CANADA 12

I think Family Medicine allows flexibility of practice, to be able to develop relationships with people, and also your communities, to be able to meet a need for your patients, and to be able to work with your patients and your community to identify that need. I think that, to me, that is very valuable and certainly one of the core reasons why I choose Family Medicine as a career.

I think certainly Family Medicine works a lot in preventative care and also working with the prevention, provides comprehensive continuing care for patients and their families. I think another benefit of Family Medicine is within the community there is being able to support not just adults or children but rather everybody from essentially from when they are born up until their last days and hours of life. I think from a community health perspective, that is very, very valuable.

I think having travelled quite a bit to different conferences, I think our patient experience that we share as a doctor is much more similar that we think. I often see other family doctors at conferences and I think that I have nothing in common with how you see your patient in the clinic; but immediately, once we start talking about the patient whether they come in for pain, or shortness of breath, or whatever the complaint may be, the approach we share is actually quite common and that approach to Family Medicine has the emphasis on relationships is something that I’m often quite surprised about when I talk and work with other family doctors from around the world. Total 2:02

CANADA 13

I guess I’ve been very privileged over the last 10 years to meet with many family doctors from all over the world: in workshops, in meetings, by email. And I think there is a couple of things that all of us have in common. I always say that although we do very, very different things, our worlds are very different; but, if you scratch under the skin, we are all the same. So, I would say, family doctors everywhere are really interested in students, they are really of course, completely fascinated by patients, patients' stories, patients' journeys. And third of all, all of the family doctors that I know, have said they really wanted to make a difference for the discipline - they love Family Medicine, they love being family doctors, and all that it can achieve. So, I’d sum it up by just saying we are really engaged in our work, and some of has to be that most of us have had to struggle. We’ve had to struggle to become family doctors, we’ve had to struggle to get our discipline recognized, we had to struggle to get students interested and through those struggles, we really are very unified, very enthusiastic and very motivated.

Total 1:20

Canada 14

I choose Family Medicine basically because of the comprehensiveness and the broadness of it. I was doing a Pediatric residency and I had already being working in the Emergency and had done a fair bit of Family Medicine before then. And for me, I missed the broadness of it, the depth and the breadth. I knew I could be a Pediatrician and focus in one area, but I’d miss the whole gamut: whether it was the palliative care, the delivering of babies, the looking after of newborns, I do like looking after seniors, doing house calls, for me that was the drive of Family Medicine and that’s why I love it.

Total 0:40

CANADA 15

[00: 00] Hello, so my name is (name) and the question is what is the impact of Family Medicine in your community.

[00: 08] Actually, even before going into medicine I always ... if I was asked what is (...) Family Medicine precisely to have an impact on the community.

[00: 17] I arrived late in medicine, I started I was 26 years old, at the age where most ended up, I was a day care educator in a health group in a day care centre in a Montreal's poor neighborhood.

[00: 30] And one of the reasons why I wanted to become a doctor was to have an impact on health, to bring health care closer to people, closer to family, closer to communities And to bring care that is adapted to the real needs finally of the families with which I already worked or with which ... I rubbed shoulders every day in the quarter where I lived.

[00: 54] So I frankly believe that family physicians are able to adapt quickly to the needs of communities, adapt quickly ... to the needs of communities and the people who make up these communities.

CANADA 16

Family Medicine is ... I chose Family Medicine because it is one of the professions in which (...) at several levels, especially at the level of contact with the human being, try to help at all ages ... let's say, from pediatrics to geriatrics, and then we're satisfied after we've helped a suffering person, at the end of a day, if we've succeeded, we feel we have an accomplished responsibility.

[00:45] This is a profession that helps a lot of people in the community, so we have contacts with the community; it’s sufficiently diversified to practice. Total: [00:59]

CANADA 17

Okay. The question is why I became a family doctor. And, I guess I’m going to talk about the first time I became a family doctor, since I became a family doctor twice [retrained from Brazil], the second time in Canada. But the real work was the first time, I was a pediatrician by background. Working in pediatrics for all of ten years, and in the second part of my career, I was looking for a certain amount of challenge. And family medicine was not really started in Brazil, so it was starting as a major mainstream force in medicine. And I thought it would be interesting, so I decided to (…?) learn a new circumstance??

It’s interesting to study a couple of things and (…?) almost as long as having a residency?

And the situation in Brazil where you can become a family doctor by working in the area and at the same time studying and becoming a specialist, eventually, by doing a test. That was my path on becoming a family doctor.

1:06 “And the whole process of becoming involved with the community was very nice, very interesting, very rewarding.” 1:14

I could say that I would recommend for young faculty members, people, students becoming doctors – it’s a wonderful experience, a wonderful specialty, especially to work in a country like Brazil where there is so much potential for improving people’s lives.

CANADA 18

My field of family medicine is actually rural practice. Rural practitioners, in many ways, epitomize an essential feature of family medicine, which is the relationships. It’s the patient-physician relationship that is the centerpiece of family medicine. And rural practitioners live in the community they serve, so they have not only relationships with people they get to know and care for as patients, but also in other social and recreational settings. Rural practitioners in family medicine, when you compare them to family physicians in big cities, they’re extended generalists. They’re providing a wide range of services and carry a high level of clinical responsibility in relative professional isolation. And because of living in the community they serve they have the opportunity to actually influence the health of the community on a whole community level in a way that, these days, family doctors in densely populated urban settings just don’t have the same opportunities.

CANADA 19

I suppose there are two stages of any medical career, one why you become a doctor and secondly why you become a family doctor. I became a doctor simply because of a pool shot. I had a decision to make for various reasons between going into a program in history, in honors history, or going into medicine, and the pool shot determined that I should go into medicine thereafter. Why family medicine? I initially went in with the idea that I would like to be a psychiatrist. And the time that I was going through medical school the stigma associated with psychiatry and society’s [?inexorable] way of dealing with mental illness was such that the only way I thought I could make any meaningful contribution to mental health was to go upstream from there. And that meant family practice. And in the interval, I simply discovered obstetrics, palliative care – the bookends of other people’s lives – and because it is in fact a front row seat in the pageant of life. And we need to be grateful for that and I have been for over forty years.

COSTA RICA 1

Well, what leads me to be a family doctor ...

The holistic vision that has,

That you can focus on the patient, talk to him, look at him, treat him, in a way completily integral.

That brings me the attention in the specialty

What is the impact of family medicine in our community ...

Well, in many ways, we are still very little in our country,

But it is newsworthy when a family physician arrives to a community or hospital,the focus he does in linking comunity with patient is worthy, because is completly different to any other speciality

And what I saw here, that we have in common all family members are passion and love in the relationship with the patient and in trying to give them the possibility to solve the reason for consultation, to search the social environment, the family context, and the psicopathology to better understand the patient's problem.

CZECH REPUBLIC 1

[00:00] Good question. Why did I choose general medicine? I think that this is the medicine that is the most close to the patients and this medicine is rich enough.

I dreamed to be a teacher and now I’m teaching future medical doctors. I always tell them “You don’t know (the disease) your patient come with, he can have different diseases” On the contrary, if you are an orthopedist you have patients with a certain type of diagnosis, this is the same with dermatologist, but generalist have a big scope of work, an extended range/choice of decision and a close contact with patients.

[00:49] I think this is a good specialization because each young doctor can find something interesting in this domain, not only for diagnosis but also for the close contact with the patient because this is the profession in which they talk a lot about patients, in which they know [the mood/feelings] of the patients, it is really very rich. Total: [01:25]

DENMARK 1

Hello there, I’m (name) from Denmark, and I’m a family physician and also a university lecturer on Global Health. I’m happy to share my thoughts on what I think I share with other family physicians around the world. I think I share the interest in everyday life: how people live their lives, the sense of belonging in a community, this thing of being able to assist and facilitate people to live their lives, not to paternalize them but just to advise and hopefully be able to see them prosper and grow. I really do think we all share this interest and also this interest in all aspects of life that there is more between heaven and earth than what is in between the ears. There are many ways of living your life, there are very many ways of facing troubles, as you do. Life is awkward and messy, and wonderful and I think that’s what we share as family physicians: interest in the universe, curiosity, and willingness or hope to be able to assist or help and facilitate.

Total 1:19

DENMARK 2

I chose family medicine because I think it’s one of the best specialties in medicine. You can still be a detective in terms of knowing a little bit about everything and figuring out who’s ill and who’s well. Also, you get to prevent disease rather than always treat which is great and I think it’s a specialty where every day you get new stories and great things happen and so I love it.

DENMARK 3:

Ok, I will talk about why I chose family medicine as my profession. I chose it, in reality, because of different things. The first was that I grow up, I was grown up in a family with two parents family physicians. And therefore, I thought I should not be a family physician, I should choose something else. But I have always been a natural part of my life so, in, when I was a doctor, I said I just take half a year in family medicine, in primary care. I felt like a fish, that I came home and could just swim. And I think one of the reasons was, is that I have always thought that it was very important for me to work close with people and know, to learn people. To know really as person in their whole life, and from that position treat their illness the best I could, and follow them all time. So that was one of the reasons, the second reason. The third reason was that I think family medicine (?) the practice has the maturity to develop itself or the time were as a doctor. You can choose different directions but you can also go deep in some special issues. You can focus, try to develop your competencies, in many different affairs. Communication but also about specific diseases, for example. But you also have the opportunity to, to develop knowledge. Because knowledge from all the specialists is not enough for our position. So, I think there is a new feeling, general practice has everything, which is what you need to live with all your whole life. Time: 1:51

DENMARK 4

(Loud) Well, actually, I knew what I was going to be when I was young.

I was a soldier first. But that was an accident. And really, I wanted to be some kind of hero. And when I couldn’t be a hero in the first way, then I applied for (hospital?) medicine, so I could be a doctor and I’d be a hero in another way. And that was why I became a doctor.

00:28 “I always wanted to be a doctor in a little village in a rural Danish environment… in --- or ---, and that’s exactly what I’m in right now. And I feel like I have enormous good relationships over my very very kind and nice community. And in that way [it makes], it has given me the meaning in my life.” 1:04

DOMINICAN REP

(Hope I got it right – somewhat hard to hear and understand)

Hello, my name is (Name), I am the family medicine official from the Dominican Republic. (It’s my pleasure?) to say that I am part of family medicine in my country. It’s the best way to find the health care of the people of my country, but in the Dominican Republic we are trying to win the fight with the government and all the public who doesn’t help. For the family practitioner can be developed and providing the (attention?) care can be developed. So, I find everyday we are better family physicians to be developed and that impact can grow in my country. Thank you. 0:52

ECUADOR 1

I am a family doctor because I found family medicine a response to reduce the inequity that exists among health users. And I also share the same way of thinking as many other doctors in the world in that we fight for equality also in the moment of receiving health services.

ECUADOR 2

Hello,

what is the impact of the family medicine in my country?

Emmm, We the family physicians are in all sceneries,

We could be managing hospitals or health centers

Or we can be in the community

And the impact is that we are the physician of the family

and not just of one sick person or patient.

ECUADOR 3

-I chose Family Medicine, because it is one of the main bases to prevent and avoid a series of pathologies that can affect the community.

- Trying to prevent illness, we are not only avoiding a morbidity in a patient but also that its environment does not be affected. Being a sick person implies that all its social environment be sick.

-Family medicine is very important, because not only focus the illness but also in the prevention, and once you get to prevent we are avoiding that all family, patients, and community lose in health and economic side.

-I think that if all doctors thought in the future, it is necessary to work to avoid illnesses, in spite of heal, world would change. Especially in the poor people since they are the most affected when they are sick, in your economic situation, because they cannot work, and also in its personal side.

- Then, I think that family medicine should be focused in the preventive aspect, and once it be focused in this, we will have a healthy population, in biopsychosocial perspective, and a more productive society with a healthy environment. There would be less expenditure in health, and people would felt better to do their physical and intellectual activities.

ECUADOR 4

(loud!!)

My story is about why I chose family medicine as a profession.

It’s a simple story. I saw a patient in a village while I was doing my internship. I went for a weekend, and then a lady, a (sixty?)-five-year-old lady, came to me to take my opinion. Because she has been struggling with doctors, of certain specialties, for the past six weeks. She finally came, with around 500 papers (lab tests and so on). And when I listened to her, I told her “look, you have to see a doctor that’s going to take care of all of your problems.” Because, obviously, she had been seen by many specialists and they didn’t …. with the diagnosis. I convinced her to go and see the GPs in the region and that’s what she did. And the (stage?) obviously from the story: she had panic attacks and with a simple treatment she was doing well. And she was back to a normal life and back to her family. That’s why I decided that being a local doctor is a lot. It’s not all about specialists.

ECUADOR 5

I think the best characteristic shared between family physicians worldwide is madness,

And I say madness because we all have something special that makes us choose this profession,

We all like and love to work with persons, family, and community

And I believe that view, that willing of service, that desire of change things is what we have in common, whatever be the place we work, the reality in which we are, we all share the love and passion to work for the community; and the desire to change things and to get better things in benefit of the persons who give trust to us.

EGYPT 1

Good morning, or afternoon, I am (Name). And I focused on family medicine characteristics in Egypt. I am pushing family medicine for my under-and post-graduate (? Candidates). I am very interested in family medicine since I was undergraduate because my school, or college, adopted family medicine in the middle east. Normally, I hear more about the community care, the community participation, working with a family. Give all the care towards the patient as for a family. How the family dynamic is affecting disease. This patient has a chronic illness, a mental illness, a specific issue.

So, I feel a career in family medicine is my own career, which I love much and give much towards my community and also towards my students who learn about how caring for the families and patients. To know the families, because as you know, diseases, especially chronic illness, can affect all the family, not only the person who has some disease. And also, the community, much family physician can take a biopsychosocial approach towards any disease. Because the disease is not affecting only organically, it will affect medical and social status of the person.

So, I hope the family medicine will be the first hope for all doctors because they (are) have the chance more and more for caring about their patients and the families.

ETHIOPIA 1

(Sounds like the first section not recorded, somewhat hard to hear throughout)

…the population is now 103 million. From this 103 million, more than 84 percent of the people are living in the countryside, and in our country the proportion of one physician is 43 for 33000 people, one physician, whereas overall from all physicians 44 percent of the physicians are located in the town. So, the countryside people, who are the majority 84 percent of the population who are tax payers, they didn’t get appropriate health care. So family medicine is allocated primary health care and it is serving the population and as the family physicians who are the gatekeepers and serving this majority of the population.

So to change our health system, to make the health system reachable to the population, give overall care or primary health care reachable to the population to answer health problems, especially involving the adolescents. The adolescents are not having care or, who is caring for them, because pediatricians are caring for their pediatric patients, gynecologists for the gynecology patients, (unintelligible for several words), and family physicians is answering all these problems. So, to change the health system of a country family medicine is a crucial point and necessary medical indication. This is why I choose family medicine and in this way family medicine can alleviate the health problems (unintelligible for several words) of the population. 2:10

GHANA 1

Hi I'm (name) from Ghana.

I'm a family physician and I chose family medicine because it's a comprehensive organistic and I like family medicine for the fact that there’s continuity of care, sometimes I get more personal relationships there’s more contact with the patient. They develop a long-lasting relationship they say from the cradle to the rocker or from the womb to the tomb. And that's the bedrock of family medicine.

And for the fact that we are more community centered and so we are able to be community leaders and demonstrate the five star - being an advocate, being a community leader, a good communicator and teacher and a researcher. That is the foundation and it is fantastic and very important and?? Total 0:54

HAITI 1

Hello,

I am (name), I am a family doctor. Currently, I work at the hospital (name) in Haiti as Associate Director of the Family Medicine Residency Program.

Why did I choose family medicine? It's simple: as you know, it is a specialty that serves as a lever to improve the health of the general population. It must also be said that it is the specialty that serves as the first line in terms of ambulatory care. Family physicians are versatile physicians and with the unique approach that is represented by the biopsychosocial approach. So, we have a particular way to approach our patients. It must be said that the approach is holistic; we see not only the patients from the clinical point of view, but we also go to the community level to provide quality care to the population. So, family physicians are not only versatile, but they are also patient-centered. And what's also interesting about the family medicine approach is that not only is care centered on the patient, but also continuity of care is very very important when providing care to patients. As family doctors, we have values ​​that are very dear to us. First, there is respect, there is competence, compassion, but also integrity and also team spirit. As family physicians, we do not have infused science, but if we work with other specialists to provide quality care knowing that we are at the center of our activities, so we provide care in collaboration with others specialties. Last but not least, I would like to add that family physicians still have an interest in social justice. Thank you.

HONDURAS 1

So, I’m (name), I’m originally from Honduras, but moved to the States to practice in family medicine. Unfortunately, Honduras doesn’t have family medicine. That was an area which I really want to get myself engaged to because of the compassion, you can create with the patient the relationship that you cultivate over the years. I start that process, I moved to the States to do my training there and now I’m a family physician and faculty with the school, therefore I have the capacity to work with my patients, get to know them, create relationships, but also transfer that information to my students and to my residents. To me, that’s what family medicine is all about. One is the connection with your community, but also creating new physicians who will take care of your patients as well.

INDIA 1

Hi, I’m (name) from India and I choose family medicine as my profession because I come from rural area in India, it’s from Bihar. Where there’s not so much, lot of attention and the rural health standards is not at par with the urban standards. So, I thought to teach family medicine as my future profession and my planned future is that to go set up my own rural clinic, in my area, and to provide affordable, quality healthcare in the people who cannot afford care in the urban area. Who cannot go to urban area to tertiary centre or secondary centre. That can come to our centres at affordable price we can provide good quality care. And our care will be more accessible to people who may not access the tertiary care situated in the urban area which is much farther from the rural areas. Thank you. Time: 1:02

INDONESIA 1

Ok, in my opinion, what family medicine can give back to my community. You know, we have general practitioners, 150,000 in my country and we have 31,000 of us specialists, and there is a gap between these two communities, one GP and one specialist. When we have a case, in rural areas especially, they have to go to the different hospital far. It’s far away, sometimes four, five, six hours, in between. So, sometimes it’s too late to help the patient when the general practitioners to not have the competencies to solve the problem. So, the people will need these family physicians, to cure them, to help to cure their diseases in first line therapy.

I believe that family physicians will benefit most people in my country. That’s why we are working on it, to make this dream come true to help more people, to help more specialists, especially in rural areas, so they can live better and cure their diseases.

01:46

INDONESIA 2:

Hello, I’m (name) from Indonesia. I am a medical doctor. And I want to be a family doctor because since I was a kid I dream of being a doctor who care for the patient and the whole family of the patient. From the kids, the parents, also the grandparents of the family. Unless you have a long relationship with my patients and give continued care for all of my patients and give them the best care. That’s why I really want to be a family doctor. And I hope the family doctor’s program in Indonesia can be realized soon and to join you from all over the world. Thank you. Time: 00:39

ISRAEL 1

Hi, my name is (name) and I am from Israel. And I am a family physician who used to work in the Kibbutz community which is a communal settlement with people at one place and one family physician.  The impact of family medicine in my community is very positive and great.  They believe that the family physician is the one who knows them, they treat the disease - doesn't treat the disease but treats them and their family and he or she will escort them in the healthcare system and take care of them even if they are in the hospital or other places. Total 0:51

ITALY 1

I chose medicine to take care of the patient in a holistic vision for a biopsychological approach. I believe personalizing medicine, patient-centered, an area of medicine which is made over medical examination, recommendation and finally of the prescription. I believe, first, do no harm. Then, the humanitarianism, the solidarity to work together for a common reason, for the improvement of healthcare. Time: 00:43

JAMAICA 1

OK, I’m (name) from Kingston Jamaica. I have been a family physician for over 30 years. As a young medical student going through med school, I found it very difficult to choose a specialty, because I found that there were so many different areas that appealed to me.

00:28 “I think what drew me to Family Medicine the most was really the continuity of care that we offered to our patients. The fact that I could develop a long-lasting relationship with my patients over the 30 years I was in practice, I saw children grow up, have their own children, and then bring their own children to me. It gave me such a sense of being able to make a difference in the life of a family.” 01:03

If I had to do it all over, I would choose Family Medicine again as it has been rich and exciting experience for me. Total 1:18

JAMAICA 2

GOOD. So, what led me to choose family medicine as a career? Well the truth be told family medicine chose me. You see after finishing medical school there were a couple things that I knew. I knew the things I did not want to do. I didn't want to do anaesthetics, I did not want to do anything with skin and I didn't want to do anything with eyes. So, what was left to be done. Well after a year or two of trying to figure out what I wanted to do. A friend of mine, a colleague of mine who had exams asked me to sit in his practice while he had to study. And there I developed my love for family medicine. Because it provided me with several options.

One I could do many things, I didn't have to specialize in skin I didn't have to do gyne, I got a chance to interact with babies I got a chance to interact with adolescents, with old people. And more importantly one of the strengths of family medicine and family physicians is that they valued friendships so I developed quite a lot of friends during the time. So, I enjoyed it! Total 1:08

JAPAN 1

Hi guys my name is Dr (name) from Japan

I've worked as a family physician in rural Japan in charge of one center with several hundred people. When I was a high school student, I was interested in family medicine because patients want to be under a doctor in the countryside and they respect me because I am in charge of all the day every day every year so I know the patients and patient history, their background.

So that's why I want to be a family physician but uh in Japan it's not familiar with family physicians so by law in my position is to promote family medicine in Japan so that is what I'd like to do in the future. Thank you Total 1:11

JAPAN 2:

Hello, this is (Name) from Japan, Department of Family Medicine Meer(?) University School of Medicine. So, these are why I choose family medicine. It’s because family medicine is brand new in Japan. I want to seek the new identity, like family medicine. I want to dream and passion, because family medicine in Japan is a kind of dream, very ideal medical field. So, I want to see family medicine with my passion. It is the reason I want to be a family physician in Japan. Time: 00:48

JAPAN 3

Well, before I worked as an emergency doctor, emergency doctor, but I changed it. In Japan, elderly people (over 65 years) was 25%. They have many complicated problems for their life so I thought it important for elderly people to manage complicated social or biomedical or psychological something. They can’t be managed as emergency, so I chose it. Yes! Thank you. Time: 0:51

JAPAN 4

Hi, I am Dr. (Name) from Japan. I am graduated from Makukura (?) medical school in Japan. I graduated from? program in which we had to go to remote community after graduation. And as soon as I graduated from the medical course I went to remote area and I worked as a surgeon. But for me, the inhabitants want a general practice and family doctor, so I changed from surgeon to family doctor. That’s why I choose family doctor. Thank you.

Time: 1:00

KENYA 1

My name is (name). I come from Kenya, and that’s part of Africa, Sub-Saharan Africa. And I have been working as a family physician since 2008. I was amongst the first graduates of the first family medicine program at (Insittution name), which is a University in Kenya, and I have been working there since 2008 when I graduated.

0:35 “I work in western Kenya, in a rural hospital, which is a primary care hospital, because currently what we call primary care is hospital-based. Since we started the program there, we have managed to have significant impact with the community because of the way we approach care.” 0:58

1:00 “Mainly, we are people-centred. We do not focus on disease, we focus on persons and their families and also the communities they come from. So, we have tended to extend care from the hospital to their homes where these people come from. And they have seen the difference in our way of practice,” 1:26 from what has traditionally been, just visits that are not necessarily centered on the person and where they come from.

The patient load from the hospital where we work has risen almost five-fold. But what is more important is the impact our practice has had in this community. They mostly have registered. They are interested in continuing to work with us, not only on matters of health but also health promotion, so we visit them even when they’re not ill and we encourage them to come and see us. And when a member is unwell, we use them to get to the family. Let’s say we get a man or a woman who has tuberculosis and we take the opportunity to get to the community and this is a fair share of what a community-oriented practice of patient care. So, we have seen significant growth in matters of raising up health conscience within the community we work with, and they have appreciated the presence of family doctors within them. 2:54

KOREA 1

Hi, my name is (Name), and I am a third year resident in (Institution name) in Seoul, Korea. I decided to choose family medicine as my profession because I wanted to be a friend with patients who need help but do not know what is desirable in their particular circumstances. The difference between family physicians and other specialists is that we do not just focus on the disease but we take some psychosocial aspects into the consideration. In addition, we get involved in almost every stage of the life cycle with the patient and the relationships between the family physician and the patients can continue in the community throughout the whole life.

So, I always wanted to be involved in peoples’ lives as a doctor and as a friend. So what also (impacted?) for me that family physicians are active in public health matters and research. So, it’s my last year of training and I hope I become a good family physician in my working. Thank you! 1:10

KOREA 2

Hi, I am (Name), I am a Korean family physician. In Korea, most family physicians are working with serious competition with other specialties. I think our quality of medical resources are the top class of the world and almost all Korean family physicians can do gastroscopies and ultrasound in their own clinic, and they love their patients, very much so, so I think they can give their patients the best medical services in primary care setting. Thank you. 1:00

LEBANON 1

I was a medical student when a relative asked me to give her a ride to the hospital. On our way we were stopped in the traffic and a female beggar approached the window of my relative. The beggar uttered her usual cache of words, “Help me, may God bless your health.” And the answer came from my relative, “I have no health. I am hypertensive, I have coronary artery disease and now they tell me that I need hip replacement.” The beggar proceeded, “May God protect your husband.” “Well my dear, I am widowed for the past 10 years”, replied my relative. The beggar here became frustrated, and she decided to continue, “May God protect your children.” And my relative said, “My dear, I have no children.” So, the beggar ran short of words. She became desperate but again she proceeded, “May God keep your wealth.” “Ha, my wealth!” she laughed. “I have no wealth. Where’s the wealth? I’m selling my property just to get treatment and cured.” [break in film/pause] “You know what?” said my relative to the beggar, “I think I should stand in your place.” Here the beggar became shocked and in a second, she disappeared. That day I realized that my relative was very depressed. She was being followed up by many specialists and despite this nobody addressed her at the psychosocial level. I advised her to see a family physician, at that time when I was in med school. And she followed my advice and she got treated and she was in good health then. This is our trade: family medicine specialists look at people globally and they address their psychosocial problems. This is a trade that is unique to us as family physicians.

LEBANON 3

My story is about why I chose family medicine as a specialty. This is a simple story concerning a patient in my village when I was doing my internship. I went on a weekend for the village and a lady; a 35-year-old lady came to me to take my opinion because she has been struggling of doctors of different specialties for the past 6 weeks. She had the file in her hand, which is around 500 papers, lab tests and so on. And when I listened to her, I told her “look you have to see a doctor that’s going to take care of all your problems. Because obviously you have been seen by many specialists and you didn’t, you didn’t, have the right treatment or the right courses.” I, I convinced her to go see the GP in the region and that’s what she did. And this lady, obviously from the story, she had panic attacks, and with a simple treatment she was doing well, she was back to her normal life, and back to her family. That’s why I decided that being a global doctor is, is an honor. It’s not only specialists. Thank you. Time: 1:26

LESOTHO 1

Hi, my name is (Name) from Lesotho. I was first introduced to family medicine in Lesotho by the Department of Family Medicine, (Insitution name). They started their postgraduate training program in 2008 in Lesotho and when they described and explained what family medicine is, I wanted it for my country, and I knew that was what I wanted to do, and from there on I never looked back. Thank you. 0:27

MADAGASCAR 1

I will talk a bit about the impact of Family Medicine in the community where I’m living now.

As defined at this international conference, Family Medicine could bring a lot since the frontline care would be ensured by those (Family) doctors.

Only by responding to the profile (of health providers) expected by the population, this is already a positive point coming from Family Medicine since the doctors that will be trained must respond to common the diseases prevailing in these communities.

Second, the most important thing is prevention, since these doctors will have a little more training in primary prevention of diseases that prevail in the community, such as infectious diseases and other community-based illnesses, and also the messages to be given to the population to avoid communicable diseases.

There is also, for instance, cardiovascular diseases or diabetes that would be well managed because family doctors will have advanced training to effectively management them at the frontline. That’s what I had to share concerning the positive results of the training of family doctors. Total: [01:38]

MALAYSIA 1

Ok Hi my name is Dr (name) from Malaysia. I'm a new family medicine specialist working as a specialist for the past three years. This is the career that I love because it's actually my passion since I was a small kid.

The main reason why I love to be a family physician is because you are able to take care of the whole family starting from the womb to the tomb. Being able to see the family dynamic, be able to take care of a lot of things and from there we are able to see the progress of the family from the beginning to the end. It really satisfies me as a doctor and I feel this is something that puts me in a situation where I am able to interact with the community most of the time. So, I think this is the main reason for me to become a family physician. Thank you Total 1:18

MALAYSIA 2

(loud!) Family Medicine has really improved the health of the communities in Malaysia. We already have 3-400 family physicians who, I feel, provide very good health care to all levels of society and in different views. At the moment, what we are trying to do … is creating a core of teachers, train more family medicine teachers, because we have so many untrained family medicine doctors. By doing this, we will be able to give our community the best-ever care for the most number of people.

MALI 1

Okay, your question is about what is the impact of Family Medicine in my community. First, I’m the dean of the medical school and the Family Medicine was introduced by the university in 2011.

With the introduction of Family Medicine program it has improved the quality of medical training in my university, first by training with new approach of competence based learning to my faculty, with faculty development in family medicine training and this has increase the quality and the capacity of medical training of my faculty who participated to the training in Canada and also in Mali.

Second, the importance of the Family Medicine program in my community, it has been the improvement of health care in rural area and with the program we have now a training centre which we call university rural health centre. So, this University rural health centre allowed the training of residents and also even premedical students in community medicine approach.

So with that, that give unique opportunity in this centre with the minimum package to deliver high quality care to the community and we have five of them now in Mali. So, it is a model we want to replicate, so that is another advantage of Family Medicine program. Total: 02:40

MÉXICO 1

Well, Why I chose to be a family physician is because I care the people, but more than people I care about the comunity and my country, And if people is healthy my country would be healthly, and I want to live in a great country, because my country is awesome,

And also I think that family doctors have lots of knowledges and they know when refer to a especialist, but once they are refering I think that you can start with prescriptions saving time to others specialists.

I think that family medicine is having impact in my country, because nowadays young doctors are thinking about making the residence but not because they want to be especialist but because they care and I think that the young people have a lot of energy to give people a good medical attention.

MÉXICO 2

The impact of family medicine in my country is very important,

Our health system is based in an 80 % in family medicine,

The family medicine system reaches practically 80% of the population,

Family physicians are in charge of the primary care, this is very important for the system, that is why family physicians are the cornerstone of our health system.

MYANMAR 1

As the general practitioners of Myanmar, there are no vocational training for the general practitioners. So, we choose one profession to train in, typically, which kind of education will help them educated. And then they provide their patients with ethical, more quality care. So, we find out that family medicine is a very good principle, just sharing among, among, among all the other family, family doctors. Among, all over the world. So, um, we found that patient-centeredness, and comprehensive cares, and continuity of care, these all are, we are practicing with all our patients, from (room?) to, from the birth to the, until the graveyard. We care all the patients. So, I like this special part. So, I choose family medicine as our main education system for GPs. Time: 1:13

NAMIBIA 1

Hi, my name is (name). I’m a family physician from Windhoek, Namibia. Why did I become a family physician? You know, for me, Family Medicine encompasses everything in medicine. I could never really see myself pinned down to one specialty. So of course, when Family Medicine as a specialty became available, that was it for me. It encompasses everything I want to do as a doctor. Now that I’m a family physician, I’m originally from Namibia, and currently I am the head of Family Medicine at the (Insitution name), which is just about six years old and we’ve just-just this year introduced Family Medicine into the undergraduate curriculum and to see how these youngsters commit to Family Medicine, the core principles that we teach, it’s just too, too wonderful. There are also Family Medicine doctors in Namibia and they have been very, very supportive. So, has the government, and so has the university. I’m so enthused by this great ideology for Family Medicine in Namibia, and the bright future, if we harness all of this­­­­­­­­ enthusiasm and this energy. Total 1:34

NEPAL 1

I choose family medicine for my career because when I got my first XXX (?). ??… Nepal, I go to many more part of my country and I saw a lot of patient with different type of problem. Like vaccines, or ??, kidney problem, artery problem and through the emergency periods there was, ah, ???. ? That I choose the practice of family medicine to solve the ???. Time: 00:50

NETHERLAND 1

I should tell you something about Family Medicine. Family Medicine is a friendship to me. Everyday, I meet people and see people and I am a very nosy person. I have human interests, so I am enjoying the stories I hear every day. It is an excellent job for the people who like people. Actually, you can and mean something for the people. As a doctor, I think you should also know you cannot always over-do something – that is the main thing.

NETHERLANDS 2

(loud!)

I took me some time to actually choose to become a general practitioner. At first, I couldn’t find what I was supposed to do. I spent a couple of years doing different things and then I said that I wanted to become a general practitioner, partly because it’s so broad. So, it’s not just talking to the patient, the adult patients - it’s the young ones, the children – it’s a real variety.

And the other thing, and I really like it a lot, compared to …(?). For example, in the emergency department you might see people for a short period of time. So, we see the patients from start to finish. So, we see patients for the first time right when they’re babies, so it helps with the puzzle to see what’s going on. And then you follow them after all the period of their illness, of their diagnosis, and you build up a – you get in touch with them. 1:08 “You walk with them, and that’s a really nice thing, I think, as a doctor. It’s one of the wonderful traits that we look for as a GP as well, and it’s the most important thing.” 1:20

NETHERLANDS 3

I’ll try my best, but it’s not my native tongue.

What made me to choose family medicine as a profession was actually the broadness of it. When I was a medical student, and I had to choose - everything was nice, everything caught my interest. I understand when you’re a family physician, you meet all the problems in medicine (how do you say it) subjects that you can find.

00:34 “And also, it’s really nice that you, in fact, come to people’s lives, to their homes, to hear their stories… yea, something they would normally keep to themselves, they share with me. And I can help them with my knowledge, hopefully.” 00:54

And that’s why I chose family physician.

NIGERIA 1

I think the first time I contacted family medicine was during my housemanship. I had started to do a rotation after finishing medical school and I think the contact with all the family physicians, and I think what stood out for me was the way they valued people, the way they reached out to people, and I realized that they do a lot to meet all of the needs and it really really caught my attention that one person could reach out and solve many problems.

During medical school what I used to seeing was one person solving one problem and then shifting off the same person to another place to solve one problem and then he gets shifted off to another place to solve another problem, but the novelty of being able to solve so many problems by one person is what attracted me to family medicine. Before then I’d been a very restless soul, I wanted to be a pediatrician, I wanted to be a community physician, I wanted to be a psychiatrist, and perhaps I can say there was a natural flare that got across, but what really got me was that role modelling, that role modelling of someone having the skills to solve different problems and having a ____ sense that reflected the things I wanted to do.

So I saw skills, I saw attitude, I saw values, I saw passion, and I saw people being successful and happy at what they do and I felt, hey, way to go, that’s it for me.

1:54

NIGERIA 2

Thank you. I chose family medicine because at the time I took the training as a family physician it was not easy for people to provide full services at a very rural level, and family medicine turned out to be that specialty that could allow me at that time and up till now to provide broad based medical care, especially to people in rural areas where I come from.

This is what informed my choice of family medicine and over the years I have practiced this specialty and I have actually moved to a level where I have been training and I have trained so many family physicians and that’s what gives me fulfillment. A good number of those physicians trained are providing services not only in Nigeria but also in other West African countries such as Ghana, Liberia and Sierra Leone, because I was trained and certified by the West African College of Physicians, which is the college that oversees the training of family physicians in the fifteen countries of the West African organization. 1:24

NIGERIA 3

(Background noise, hard to understand)

My name is (Name), I’m a family physician from Nigeria _______. My interest in family medicine dated back to my youthful age. So initially I was born in a poor district ____ so I went to, in the primary school days we often see graveyard of small children _________. So, I consider it normal for children to die very young age, ___ funerals_____. So later on, in life, when I get into medical school, particularly the later years I want to realize that all these diseases that kills these children are preventable, that those children are not actually destined to die. And I looked the pain, the agony, trouble, _____ that these families have gone through, you know, the women give back so many children trying to think that a few of them will survive, (and many wives?).

I looked at, considered family medicine as a discipline that would enable me to understand the family needs, the structure, the impact associated with, upon their health and illness. So that’s what stimulated my interest in family medicine. I ___________ because ________________go to treat those children and in addition______ that led to, that organized programs in villages, small communities and the feedback has been wonderful. So, I think I helped and I made a fulfilled mission. 2:14

NIGERIA 4

I chose to become a family doctor because I have hand-down on everything, in everything, I mean that I don’t want to focus on bones or surgery, but I have to do every other thing, so that I wouldn’t get bored along the line. And apart from that, I realize also in Family Medicine, I can give my patients comprehensive care in addition to that, I can go their homes and give good health services. Family Medicine provides rationale, wide care, check all the symptoms, diagnose the disease and keep the patient fresh. As a Family Physician, I can actually care most of the patients and not have to refer all of them until I feel necessary and that is very interesting to me. I am never worried to not have taken a specialty.

NIGERIA 5

I chose family medicine as my profession because if the families are taken care of a society will be better off. And most of the diseases and the sicknesses in the community, or in the native (?) family. And if the family are well taken care of, we will have a healthy community, and we will have a healthy nation. Many of the current diseases we have been taking care of are family level, before they get to the specialist stage. Time: 00:49

NIGERIA 6

Yes, I went into family medicine after medical school, and realized that my community is filled with morbid conditions that only a broad-based healthcare provider can tackle. Therefore, after medical school I recognized that only family medicine will give me all of the skills that I will need to tackle most of the health problems in my community. And this includes communicable diseases and non-communicable diseases and mental health disorders and so on and so forth. And I found that as a family physician I have been able to fulfill this dream of being able to provide a broad-base care. Time: 00:51

NIGERIA 7

Family medicine as a practice, I chose to do – pure family physician – because family medicine is actually the aspect of medicine that deals closely with the patients. We have immediate and direct contact with our patients and most places you treat 80% of all cases and you act as the gatekeeper in medical practice. You sort out the cases and treat 80-90% (it depends on the part of the world). And then in many roles you sort out the relevant specialists who then give you feedback. So not only does the family physician manage the patient, he is primarily the owner, the doctor, of the patient. So that is just why I love to be in the main, you know, the body, the doctor, the practice that actually owns the patient, that sees most of the cases in medicine. And in family medicine, you know, we don’t segregate. You don’t sit them down outside and say, you know, the next case is going to be a urologic case or a gynecological case or a pediatric case. We just sit there, as cases come in, we sort them out. So, we are more or less like the super doctor, seeing 80% most times of the 100% of sick people. We sort them out, send them away, maybe 20% for other doctors to manage. And even though the other doctors, they still they turn to you about their (?) specialty. So primarily speaking you are the real doctor to all the patients. Time: 1:36

NIGERIA 8

A family physician, like you know, is like a medical general, like a general medical practice. The … The people that calm(?) the patients. And as a general physician, or family physician, 90% of all the cases we see in medical practice they can be solved. 90% of cases, or problems, can be solved by a family physician, which leave only about 10% of very significant infection for the so-called specialists. I mean we use a biopsychosocial approach to see almost every sickness. (?) sickness is to see within the context of the whole of the patient: biological, physiological, environmental, cultural context, (?) context. Honestly, we’re docs! Family medicine, head of my heart ☺ Time: 1:04

PARAGUAY 1

I believe that a characteristic of family doctors all over the world is we have an integral and holistic view of patients; we can do interventions in all aspects of patients, not only in bio-medical aspect, but also of social and familiar aspects.

We do a person-centered medicine and not only in the illness.

This opens to a kind of medicine which was lost in some places, that is the person-centered medicine, what gives us the possibility to make individualized interventions for each patient, according to its social, familiar, emotional, physical, economical condition.

And also, the family doctor is the entrance door of the health system. In Paraguay we are starting with primary care, it started in 2.008 with first seven units of family health, and currently we have 107 units of family health and the idea is to reach 700 units of family health to cover all population, this makes a change in social prevention in the country.

As we know, having this units of primary care, makes a better attention for people, allows physician to interacting with community, and makes medicine and attention more accessible.

PERU 1

Hello, my name is … (I cannot understand the audio)

I am from Peru, Lambayeque

About what makes me chose family medicine as a career, I am a person who comes from the community then I identify a lot with it

My passion is serve people, and family medicine allows me to get to the persons from all social status, and to work with them gets me so pleased.

PERU 2

Firstly, a very kind greeting for everyone here, at the World Conference of Family Doctors,

I understand that Family Medicine is growing up in all countries

And it is important to strengthen the specialty, in order to may improve efficacy and cost of the treatments

Seeing that the tendency of medicalization of the health exists and it is known that it is not convenient for primary care

So, I think we should improve all over the world the primary care

Because of the many benefits that brings to general population health.

PERU 3

What led me to choose family medicine as a profession is because it has the values of primary health care.

The impact of family medicine in my community is not giving results yet

We are in a change process

What I can share with my colleagues from other countries is that we must fight for the change of primary care.

PHILIPPINES 1

Hi, I’m (Name), I’m the Philippine representative for the (Institution name). Now I’m here to tell my story of what that means to choose family medicine. I want to choose family medicine because, first of all, it addresses all ages, it’s more people oriented and I love how we address the concerns of a lot of people. I think that will be all. 0:38

PORTUGAL 1

0:05 - I chose Family Medicine because it tends to see the patient in a broader perspective

0:15 - in a global context.

0:18 - An interesting idea is to treat health problems preventing them from happening.

0:23 - It’s probably the more effectively way to our future (health system),

0:29 - because we’ll save economic resources

0:32 - and bring a better quality of life to our patients.

0:35 - Family Medicine put it all together

0:38 - And reaches this multifactorial scenario,

0:42 - helping us to do a better work.

PORTUGAL 2

0:01 - Good morning, I’m a Family Physician in Portugal

0:07 - It’s one medical specialty that, when it was the time to choose which residency I’d like to do, was in my list of options.

0:17 - I cannot say that it was my first option

0:23 - but, in fact, after having more contact with Family Medicine, I wouldn’t change for any other medical specialty

0:33 - being a Family Physician is completely different than others hospital-based medical specialties

0:41 - We can see the patient in a comprehensive perspective, in the community where they live

0:47 - And I realised that it makes a huge difference

0:56 - because we can, by treating one patient, treat the whole family

1:01 - and reach in fact the whole community

1:03 - We have a way of looking at things that is different than that from our colleagues in hospitals

1:10 - and it makes the whole difference:

1:13 - “We treat the patient and our colleagues (in hospitals) treat diseases.”

1:20 - We can see babies, kids, teenagers and adults

1:30 - and this holistic view is very important.

1:34 - In my perspective, all of it makes a Family Physician.

PORTUGAL 3

0:02 - When I was a child I wanted to be a scientist

0:06 - I was 5-6 years-old and I wanted to be a scientist.

0:11 - soon I realized that being a doctor I could make my dream come true,

0:18 - by simultaneously treating people and being closer to science.

0:26 - so, at the age of 6-7 years-old that I chose to became a doctor.

0:31 - Later in my life, things started to change

0:35 - and I started to think about what to do.

0:39 - and when I finished medical school and had to decided what career I wanted to do

0:42 - I worked for a while as a generalist with no specialization

0:46 - And later I decided to do my training in Family Medicine

0:58 - I’m happy as a Family Physician, as a mix of healer and scientist.

QATAR 1

So, we’re ready. The question I think I’m going to answer is why I chose family medicine. It’s more because of initiative for (?) because I was actually going to - becoming a pediatrician – but since moving to the UK, I sort of realized that I actually enjoyed being more generalist than actually specialist. And the other thing that caused it was having work-life balance. I think now having been in general practice for over fifteen years, I think I made the right choice. I wouldn’t change doing that, because it is something, I’m passionate about, it’s something worth – actually make a difference. And I know that, you know, 1:04 “the future of health care lies in primary care.” 1:09

RWANDA 1

Since I graduated as a medical doctor, I practiced for twelve years as a GP in a district hospital in a rural area (remote area) where I would see many issues to provide quality care for our patients. And one of them is that we used to refer cases that we couldn’t manage, just by lack of expertise, lack of direct skills to do this.

0:42 And sometimes we would refer the patient, let’s say with having postpartum bleeding, who just died on the way. Just because I am unable to do a hysterectomy. And that’s why I realized I still missing some competencies, some skills which would help me to do more for my patients at some district level. That’s why I thought Family Medicine. 1:12

Reading about Family Medicine, I realized that ‘yes, that’s the answer for me’ in terms of my knowledge and my skills in terms of clinical work but I got more than clinical work.

1:27 Actually I realized I can be more than just a clinician: an advocate, a manager, even a better communicator. I’ve been getting more than expected from Family Medicine. 1:38

For those who want to join this exciting specialty, I would say just come and join us. And you will not be deceived, you will be happy with this. Thank you. Total 1:51

SAUDI ARABIA 1

So, my name is (name), and the reason why I choose to be a family physician is because I wanted to do something that is rewarding and that’s constantly helping individuals and that’s certainly patients.

0:20 “So when I was doing my internship [um], and rotating among all the specialties like: internal medicine, surgery, etc., I just felt that sick patients continue to be sick and that there is really no way of me helping them to the point that I was hoping for. Whereas, in Family Medicine, most of the patients that I was able to see, I was able to give them either a [like] short-term or a long-term management plan which at the end I could see the effect, I could see the rewarding aspect of it. “ 0:55

0:56 “The reason why I wake every day in the morning and feel energetic and enthusiastic about going to my clinic is because it’s a very rewarding field. With each patient you get, you listen to a new story; you can never tire of what will be the next patients come complaining of. So, it’s this sort of excitement part [loud background] - meeting new people, meeting your same patients still, but coming with different concerns or a different story to share with you. I love very much the continuity aspect of Family Medicine and I will always be proud to be a family physician.”

SLOVENIA 1

So, (name) said that she decided to become a family medicine doctor as she was a child. Because this was a really nice thing; this was really important for her.

She was not expecting to work in a hospital. She just liked to work in a field, in a different environment. Outside, with the people.

1:04 “What she likes the most about family medicine is working with people. Having the physical, and the first contact, with people. And having that actual connection with a patient.” 1:15

And that’s it, and it’s also the most important thing in family medicine for her. Thanks.

SOUTH AFRICA 1

I’m (name) from South Africa. I practice down in south of South Africa, 400km from Cape Town at (Insitution name) in Georgetown for the last 16 years. I want to just share what I think I have in common with other family physicians around the world.

I think a few things come to mind, just from this conference, is that you talk about patients, and patients often comes up in all our presentations, in lecture and talks. The …(?) to that is to keep the poorer I think I notice at this conference, members around the world is a great emphasis on giving for the marginalized poorer that particularly gets medical health and the impact to that again is the passion that people have for caring that there is evident at this conference there is something alive, an energy, that people are still excited from the young student, right to the people who have been practicing a long time. I’ve been in my practice for 16 years, in government practice, and I still sense that passionate care and I still see it around here and I think we find it in each other. People haven’t gotten into a rut. So that’s probably it: the patients, the poor, and the passion for them.

SOUTH AFRICA 2

(loud – and she talks more about her non-FM job than FM)

Hi, my name is (name). I’m a family medicine practitioner from Cape town in South Africa. And I work in primary care clinics. And implementing evidence-based programs in clinics in Sub-Saharan Africa.

The impact of family medicine is substantial – huge in government primary care clinics. We work alongside our colleagues - our nursing colleagues, --- colleagues and others. And we work together as a team unit, ---- to ensure that we apply evidence-based medicine. We work as an integration function with managed care as simply as we can. We share the information with our colleagues. For all of us, so that’s my mission and passion. Doing something to improve primary care services right across Africa.

00:58

SPAIN 1

To the first question, what led me to choose family medicine as profession ...

I would say that, it led me to the contact with the person, seeing the person as a whole, the humanism, to be referent in medicine to that person as I am his health agent, the person who coordinates all his problems an that helps him to choose other specialist when he need it.

In summary it helps me to choose, the proximity and a whole vision of the person.

SRI LANKA 1

Okay, so I choose family medicine as a profession in Sri Lanka because I want to serve my community. There are a lot of family doctors in Sri Lanka but they are from the ______. The (laymen?) they go to the physicians when they are really, really bad, actually, when they are really in very difficult _______. But in the family medicine in Sri Lanka it’s okay, so where I can serve the country, mainly in the prevention of mainly the non-communicable diseases in Sri Lanka. Okay? 0:48

SRI LANKA 2

I chose family medicine as a profession because I think it is an important area of medicine. Most persons you meet need a family doctor. So, I think that’s the most important area to promote in medicine.

SWEDEN 1

Hi, I am (Name) living in Sweden, working as a family physician, and I am a specialist since seven years ago. Before that I was studying physiotherapy in Stockholm, and one of the main reasons I chose the family doctor is that it is the compact of all the disease which am existed and I was also interested in musculoskeletal disease which is also included in family physicians. So, it’s a very wide combination of all the diagnoses and we meet all the categories from childhood to old people. So, it’s very interesting, I recommend my colleagues, my young colleagues in the world and Canada. 1:06

SWITZERLAND 1

When I had to choose what I wanted to do in my life, where I wanted to give most of my time, because work is a place where you give a lot of time, I remember it was a little before the beginning of enrollment in university, I asked myself a little about what was important to me, with whom, with what I wanted to work.

I had relatively easy school, so I thought I have to go for a university, I really wanted to get to the bottom of things, to take a time apart to think.

That was the first thing, and then the second was to ask myself what I was looking for in my work, what I needed and I think regular human contact was something I was looking for.

Then I had several ideas that came into me, there are several (...) faculties, that came out: faculty of psychology, faculty of theology; The faculty of medicine was in fact the one that offered me the most possibilities after the university.

So, I started my medical studies, I arrived in this extremely technical world. In any case, in Switzerland, the world I know, these are studies that are much given by specialists, so I arrive in these university studies, extremely technical (...) – maybe not too centered on patients.

Little by little, with the internships, I realized that what interested me the most was the patient in his entirety, it was not only an organ, it was not a system, it was really about who is in front of me, how the meeting between us will bring benefit to him, certainly also to me, in the (...).

What interested me was not only a single person but also a person in his community, knowing what makes this person that way, what makes this community work in such and such a way, what community health problem they encounter.

So I have to say that having all these questions in mind, it was quick enough to tell myself Okay I want to become a general practitioner, a family doctor, because that is where many of my interests and my goals can meet, both quite advanced scientific and technical training with many human encounters ...

On a day I do not know how many patients I will see, I still have a piece of training to do before I have my office but (...) certainly dozens of people a day and the scientific technique, meeting people, and being able to have a concrete impact in the community are really things that are extremely important to me. Thank you

Total: [02:53]

TAIWAN 1

Actually, I want to share with my patients why I wanted to be a family doctor and it is because as a family doctor, I learnt from different specialties, such as skin problems, heart problem, chronic problems, so that I can give comprehensive care. That is how the medical care system is.

We have different specialties and if you go to heart specialist, he would carry cardiac tests, and if you go to chest doctor, he may say let’s do chest x-ray, but if you go to family doctor, he will give history taking and then decide the cause for the heart pain, which may be due to bad sleep, originated due to lack of sleep or bad mood. This is precious thing about being a family doctor. You give comprehensive care and also focus on patient. This is called *patient centric care* – because everyone has different belief and we should try to make them understand the treatment and medicine they use, and what they think about medicine, they are taking. So that the patient takes better compliance about the drugs and medicine they take.

TAIWAN 2

So, I’m going to tell you why I chose family medicine as my profession in medicine. So, when I was an intern, I did a lot of rotations but I found that family medicine was the one that I really wanted to be, because it had a lot of different interactions with all kinds of patients.

So, I work in a hospital that’s solely for veterans but at the same time we also reach out into the community, such as the schools from the kindergarten to the university students. I gave a lot of lectures and I went to the villages, so you know, the life as a family physician is really exciting and it has a lot of colors. So that’s why I chose to be a family physician. 0:58

TAIWAN 3

When I am a medical student, I like to join the activity in the rural area, the medical education and the medical service for the children in the rural areas. That’s what I really liked when I was a medical student. And when I finished my medical education in the first year, I joined the internal medicine training. After one year of internal medicine residency I have to go into the intensive care unit and I think, it’s not what I want, because I work with a lot of machines but I not work with the people. So, I try to rethink about my future career and I change my career to be a resident of family medicine, so that’s why I become a resident of family medicine. That way is the best way that I can have more chance to have life with people, to say something, to understand something, to be friends with others. That’s what I like. 1:08

THAILAND 1

My name is (name), I am a Family Physician. I am working in Siachen Hospital, south of Thailand. According to the requirements of the family medicine in my community as a family doctor, after having studied in USA for 10-years, I came back to my community to work as a family doctor.

A family doctor differs from a normal doctor in a way, he has a continuity of care with the patients, to see the patient in a holistic character, this is essential. For example, in stroke patients, we have continuity of care of the patients. I have to provide quality health care to the patient, which is very impactful to the community. And I am proud to be family physician.

TUNISIA 1

I’m not a family physician; I’m a pediatrician and dean of a faculty. I’m sure that Family Medicine is one of the key elements to the realization of the goals of the right to health, equity and universal health care.

So, within my faculty and my country, the social and regional inequalities that have been highlighted in recent years have prompted us to work together, the faculties of medicine and the ministry, and convinced us all that one of the most consensual approaches was to value family medicine particularly by directing training towards priority and essentially primary health needs.

So what impact did it have? The first impact, once the integral curriculum of family medicine has been put in place, has been to review all our curricula on the basis of priority health needs and this has changed the inequality and culture at the level of education.

The impact toward our communities was really very important because we favored and prioritized problems by region. Valorizing the frontline and the priority needs gave a very favorable impact, given the doctors of first consultations, the doctor who is closest to the citizens.

We are convinced that the actual valorization of the frontline through family Medicine will allow us to progressively reduce these regional health inequalities and even will consequently lead to good health nationwide.

TURKEY 1

I am a Family Physician from Turkey, my name is (name) and I am the founder of the Family Practice in Turkey. I have been working as a GP Family doctor approximately since 30 years in Turkey. I like Family Medicine because it enables to help the people of their health issues comprehensively, so I chose Family Medicine instead of any other specialty.

I have experience in primary care and from time to time, we arrange to organize various associations and federations. Recently we formed a foundation – Turkish Implementation Research Development Education Foundation.

I have been working at my family health center in (Institution name), Bursa, where I am Associate Professor of Family Medicine.

It is very important to discuss and share all the details of the complex issues of primary care along with other countries together. Finally, the important point is to improve a good model with high standard through-out the world. I thank the Canadian Family Physician and Canada to give this big opportunity and hopefully, we will construct a good model of Family Medicine with Canadian participation.

UGANDA 1

So, we will talk about why I chose family medicine. And I think my choice of family medicine as my career originated from my first place of work when I finished medical school. Because I went to work in a general hospital which was in a very remote area at the time. It’s still rural anyway. So, when I reached there, I found there two doctors. One was a generalist, but no postgraduate training and the second one, who was actually the hospital director of the medical school there, had postgraduate training. At the time, I was working like any other junior doctor. But when I saw his work, I was impressed by what he was doing. (And I) because we would do everything. We would treat all our patients: medical, surgical, pediatrics, obstetrics, everything, manage surgery, manage (****) care.

1:06 And we are actually doing everything. And we referring, if not none, very few patients. So I asked him ‘you are doing everything’, and I had come from a tertiary hospital, where seeing patients was compartmentalized in terms of specialization. So, if you’re in a medical ward, and somebody has a wound, you have to refer to a surgeon. 1:30 That was the kind of training.

But when I saw what he was doing, I was impressed. So, I asked him ‘how can you do everything?’ and he told me ‘yeah, I am trained to do everything’. So, I said, ‘what were you train in?’, he said ‘I trained in Masters in Medicine, in Community Practice.’ At the time, because it had just been changed from Community Practice to Family Medicine.

Then I said, ‘I think this is what I should do’. So, but the issue was where do you do it. So, I asked him ‘where do I do it from?’ So, at that time, he had done his training from (Institution name), but the program had sort of slowed down. So, I was told that (Institution name) was opening a program. And I went and found out about the program and that’s how I ended there. So basically, briefly, that is my story about how I actually am in Family Medicine. 2:20 And I think I have enjoyed being a generalist. I never get bored because I see a variety of patients. Nothing bores me in clinical medicine. 2:30 Total 2:30

UGANDA 2

I wanted to become a family doctor because of my previous experiences. I worked in a clinic which was in a health centre, which was a community health centre, and had quite a number of patients with different diseases. But many times, you treated the patients you realize that they only wanted medicine, they needed a lot, like talk to them about their illnesses are, what is the meaning of the illness, maybe things like what can they do to prevent. So, but, I didn’t see this was going to give me that, and I liked family medicine. So, family medicine was a good, (Name) allowed me to help my patients understand their issues. 1:06 (One phrase at the end unclear)

URUGUAY 1

What led me to choose family medicine as a profession…
Because family medicine specializes in people, it can see human beens in all stages, and along all his life and it can see the healthy and sick humans
Accompanying the most beautiful moments but also the most painful ones
And I think it is an speciality which makes me happy, and I see that people like it.
What is the impact of Family Medicine in my community?
In fact, my community had a transformation since our family doctors are working on it
We had a closed health care center, but now is open and people attend
And I think the best traits I share with others family Medicine doctors of the world… happiness for what we do, love for the people, and this feeling of accompanying all the family along his life.

URUGUAY 2

About the question, what is the impact of family medicine in the community I work.

I think it is mostly the contact with people and families,

With the posibility of really accompaining what the people are living at that moment

And to approach what people live in their homes, families,

I believe that the impact is not only between the family doctor and the community, but also with the rest of the healtcare team, and others that work in that territory, and working that way we get the positive impact

Building together the collective knowledge

Giving a more humanist view of the medicine, that makes the difference.

URUGUAY 3

What led me to choose family medicine as profession,

I think is the only way to follow a family, a patient or a community along all his life,

I think there is no other speciality that alow to develop the skils to treat since the begining of the life, gestation, with pregnancy control, with the birth,

in our country we have the posibility in our formation to may atend a delivery, well child visits,

the relationship betwen families, how families are in the comunity,

how adolescents develop, when they become adults, how they can form new families,

then the apparition of ilness in elderly people, the nets can generate with elderlys,

and also to acompaing the dying process

US 1

So the question is why I chose Family Medicine. When I first got into Medicine, actually it wasn’t in college. It was after I finished college, studying Astronomy and Physics, with no intention of going into Medicine, but before I was going to do graduate work in Astronomy and Physics, I decided I’d better go learn something about the world. So I hitchhiked around the world for a whole year. I went through Asia and Africa, not really with a set itinerary, I had friends to see along the way, but basically, I spent almost a year travelling on $4,000. So it was shoestring travel and I hitchhiked, took boats, took canoes, took buses, all public travel, stayed in the cheapest little places I could afford to stay. During that time, I got the idea that practicing Family Medicine or being a doctor in the developing world would be very rewarding.

Being a doctor in the US never really caught my eye. I don’t have any doctors in the family; I didn’t have any doctor role models; to me, growing up in Los Angles, a doctor was a guy in a black Porsche in Beverly Hills who wasn’t very happy, probably divorced – they made money, but I didn’t see a lot of social good to it. After traveling this year after college, I was 22 years old, I thought: wow, being a doctor is a passport to work anywhere in the world, I could help people, I would be needed, I would have a skill, the ultimate skill you could use anywhere. It’s better than learning one language, if you could learn the language of Medicine, it would be very useful. So that was my initial idea that might have been a little naïve, as it is hard to go from country to country without knowing the cultural context, but that’s what got me interested in Family Medicine. I say Family Medicine because you know, while travelling in Asia and Africa, this year of travel was mostly Asia and Africa, not Europe, you look around and clearly, half the population is either kids under age 15 or women giving birth to those kids. The idea of doing anything that didn’t include women, their reproductive healthcare, pediatrics, would have not been a good medical career. So when I started medical school, I already knew I wanted to be in Family Medicine, it wasn’t go to medical school and decide what to do later, to me, I had to take care of children, and I had to be able to deliver a baby. So either I did Peds, or OB, or Family Medicine. And then, those three, screaming kids all the time is a bit much with me, and I realized also from talking with my OB colleagues, my residency in America, I realized that OB that’s suppose to be the best for learning women’s healthcare, primarily OB is a channel for obstetrical surgery. Potentially OB is a surgeon who specializes in gynecology surgery and I was not interested in surgery very much at all, so pediatric healthcare important, yes, but do I want OB surgery no. It just was a natural to choose Family Medicine. So Family Medicine kind of came out from this trip while travelling the year after college. That’s the story. Total 3:10

US 2

Hi, I’m (name). I’m a family physician from the United States of America, I live right in the middle of the country. I grew up in a small area, a town of about 5,000 people. To me, family medicine was a natural choice, although I didn’t know it right at the beginning. I wasn’t sure, but the image and view and perceptions that I always had of what a doctor was a family physician. I just didn’t know yet until I went to medical school. I had some great mentors in our Family Medicine interest group and the turning point for me was when I attended a Family Medicine interest group about my second year of school and they had family physicians doing all kinds of different things: serving the communities that they lived in, and I really liked the idea that they were doing what was needed in their community and I thought that this was the job for me. Total 0:51

US 3

(very loud and she talks more about her job than FM – not sure she is one?)

Hi, my name is (name). I work with ---

For me, family medicine is critically important to the area I work in, which is the global health community, and the TB community. We work to find tuberculosis ending by 2030.

And what we’ve realized through our work over the last twenty years, as our search continues, is that TB is a family disease, a community disease. And the only way to make an impact is to work with people working as closely to those affected as possible.

Family physicians can make an incredible difference in Public Health authorities, in working with NGOs and in working with individuals who are affected. Both the people who have the disease and their family members. Some of the things that we’re most concerned about is the huge costs the patients bear, not from direct payment costs, but income loss and social costs – the stigma and marginalization that comes with TB. Family physicians can both help people gain the best medical care but also identify the social services they need and identify the needs of their family for prevention of TB. And for prevention of the other health problems that lead to TB. So for us, it’s a no-brainer. Family medicine is essential for public health and for ending TB.

US 4

I chose family medicine because, despite going to a medical school that didn’t have a department of family medicine, I was fortunate to find some mentorship from some really amazing family doctors who really showed me the breadth of family medicine, the opportunities to serve patients and serve communities and engaged communities in a pretty unique way by have certain continuity and relationships with patients. And I think that family medicine also provides as a specialty really certain unique opportunities to engage in advocacy with and on behalf of patients. Whether it’s in the exam room or it’s you know in the halls of congress, in the United States, in Washington D.C., that sort component in a specialty was really important to me and really drew me in.
